# Steroid Receptors in Breast Cancer: Understanding of Molecular Function as a Basis for Effective Therapy Development

**DOI:** 10.3390/cancers13194779

**Published:** 2021-09-24

**Authors:** Wojciech Kowalczyk, Grzegorz Waliszczak, Robert Jach, Joanna Dulińska-Litewka

**Affiliations:** 1 Chair of Medical Biochemistry, Jagiellonian University Medical College, 7 Kopernika St., 31-034 Kraków, Poland; w.kowalczyk@student.uj.edu.pl (W.K.); grzegorz.waliszczak@student.uj.edu.pl (G.W.); 2 Department of Gynecology and Obstetrics, Jagiellonian University Medical College, 23 Kopernika St., 31-501 Kraków, Poland; robert.jach@uj.edu.pl

**Keywords:** breast cancer, steroid receptors, estrogen receptor, progesterone receptor, androgen receptor, glucocorticoid receptor, mineralocorticoid receptor, vitamin D receptor, molecular function

## Abstract

**Simple Summary:**

The knowledge we currently possess on the molecular function of steroid receptors in breast cancer is incredibly vast. New research in the field is constantly emerging, including studies focusing on potential therapeutic application of steroid receptors other than estrogen receptor which already serves as a crucial therapy target. Therefore we believe that it is necessary to regularly summarize the data on this topic. The aim of this review is to provide breast cancer researchers with a clear explanation of the complex nature of steroid receptor function, including the most up-to-date information, in order to support the effective development of future hormone therapies.

**Abstract:**

Breast cancer remains one of the most important health problems worldwide. The family of steroid receptors (SRs), which comprise estrogen (ER), progesterone (PR), androgen (AR), glucocorticoid (GR) and mineralocorticoid (MR) receptors, along with a receptor for a secosteroid—vitamin D, play a crucial role in the pathogenesis of the disease. They function predominantly as nuclear receptors to regulate gene expression, however, their full spectrum of action reaches far beyond this basic mechanism. SRs are involved in a vast variety of interactions with other proteins, including extensive crosstalk with each other. How they affect the biology of a breast cell depends on such factors as post-translational modifications, expression of coregulators, or which SR isoform is predominantly synthesized in a given cellular context. Although ER has been successfully utilized as a breast cancer therapy target for years, research on therapeutic application of other SRs is still ongoing. Designing effective hormone therapies requires thorough understanding of the molecular function of the SRs. Over the past decades, huge amount of data was obtained in multiple studies exploring this field, therefore in this review we attempt to summarize the current knowledge in a comprehensive way.

## 1. Introduction

In 2020, breast cancer became the most frequently diagnosed cancer worldwide, regardless of the sex. In women, it accounted for almost a quarter (24.5%) of all cancer cases and remained the most common cancer-related death cause [1]. Steroid receptors (SRs) have long been known as key players in breast cancer pathophysiology. Over the past decades, they have been extensively studied but still remain a major subject of breast cancer research.

**Figure 1:**
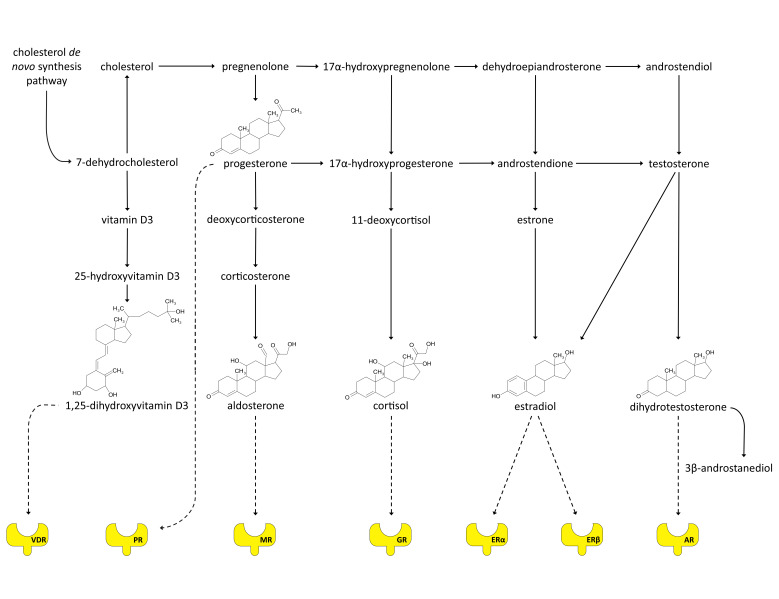
Structure and synthesis of ligands for SRs and VDR. Structures are shown only for steroids of highest affinity to their target receptor [4,6]. Since ERα and ERβ are encoded by different genes they are shown separately. Solid lines should be read as “is metabolized to” and dotted lines as “binds to”. Illustration created using elements from Servier Medical Art https://smart.servier.com/, reproduced under Creative Commons Attribution 3.0 Unported License https://creativecommons.org/licenses/by/3.0/.

Steroid receptors are polypeptides which belong to a superfamily of so called nuclear receptors—evolutionarily and structurally related cytoplasmatic proteins able to translocate to the nucleus where they act as transcription factors (TFs) to modulate the expression of certain genes [2]. The family of SRs comprises estrogen (ER), progesterone (PR), androgen (AR), glucocorticoid (GR) and mineralocorticoid (MR) receptors [2], all expressed in healthy human mammary gland as well as in various types of breast cancer (Expression Atlas https://www.ebi.ac.uk/gxa/home [3]). Additionally, our review also covers vitamin D receptor (VDR), the ligand of which is a secosteroid, 1,25-dihydroxyvitamin D3, synthesized from 7-dehydrocholesterol, making it closely related to classical steroid hormones which are cholesterol derivatives (Figure 1) [4]. VDR action considerably affects the function of breast tissue and plays an important role in breast cancer [5]. Membrane receptors for steroid hormones are not a subject of this review.

Nowadays, breast cancer endocrine therapy is based on targeting ER. The major drugs in use are tamoxifen (selective estrogen receptor modulator—SERM), acting mostly as an ER antagonist, and aromatase inhibitors (AIs) which repress the synthesis of ER ligands by blocking the conversion of androgens to estrogens. However, according to ClinicalTrials.gov (accessed on 30 August 2021), there are also multiple recently completed or ongoing clinical trials investigating drugs targeting SRs other than ER for breast cancer therapy. These studies include e.g., NCT04738292 (onapristone—PR antagonist), NCT02651844 [7], NCT05016349, NCT01138553 (mifepristone—PR antagonist), NCT04947189 (seviteronel—androgen biosynthesis inhibitor), NCT01990209 (orteronel—androgen biosynthesis inhibitor) [8], NCT03383679 (darolutamide—AR antagonist), NCT00637897 (paricalcitol—vitamin D analog) [9] or NCT01708798 (eplerenone—MR antagonist; for cardiotoxicity prevention) [10]. Most likely, in the near future further drugs targeting these receptors will be tested. Additionally, there is a possibility that novel therapies aimed at various proteins which interact with SRs will also be designed. Therefore, here we attempt to present the complex aspects of SR molecular function in breast cells, including an in-depth description of PR, AR, GR, MR and VDR and their vast interactomes, understanding of which is crucial for developing new approaches in breast cancer endocrine therapy. Our review covers both basic aspects of SR function as well as most recent discoveries in the field.

## 2. Molecular Function of Steroid Receptors—Common Features

Steroid hormones, ligands for SRs, are synthesized in a process called steroidogenesis (Figure 1). Steroidogenesis occurs mainly in adrenal glands and gonads but certain reactions, especially the final conversion of testosterone to dihydrotestosterone (DHT) or to estradiol (aromatization) can take place in many tissues which are often at the same time steroid hormone targets [6]. The cholesterol utilized in steroid synthesis is mainly acquired from serum lipoproteins. Some fraction of the compound is stored in cytoplasm as esters from which it is released by the cholesterol ester hydrolase activated by luteinizing hormone (LH) or adrenocorticotropic hormone (ACTH). In ovaries, the release of cholesterol and its conversion into pregnenolone is promoted rather by LH than ACTH (as opposed to the adrenal glands) [6].

**Figure 2:**
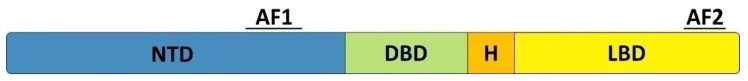
Schematic illustration of steroid receptor structure. NTD—amino-terminal domain, DBD—DNA-binding domain, H—hinge region, LBD—ligand-binding domain, AF1—activation function 1, AF2—activation function 2. The diagram does not show the exact length proportion of the domains because it differs between distinct SRs.

Steroid receptors share a common ancestry and therefore bear a close structural resemblance to each other, as all consist of four main portions, namely the C-terminal ligand-binding domain (LBD), DNA-binding domain (DBD), hinge region and amino-terminal domain (NTD) [2,11,12,13,14,15] (Figure 2). Each SR contains also two regions called activation function 1 and 2 (AF1 and AF2) which lie within NTD and LBD, respectively, and are crucial for regulation of gene transcription. Two zinc fingers are located in the DBD [2].

Canonically, the SR signaling mechanism involves ligand binding, dimerization, nuclear translocation and interaction with DNA. Prior to hormone action most SRs stay in cytosol bound to chaperon protein complexes [2,16,17]. Those comprise Heat-shock protein 70 (Hsp70) which is first to recognize and bind the SR, Hsp90—the main chaperone involved and Hop (Hsp70–Hsp90 organizing protein) which facilitates the transition between Hsp70 and Hsp90 systems. Additionally, a variety of proteins serve as Hsp70 or Hsp90 cochaperones. Namely, Hip (Hsp70-interacting protein) and Hsp40 act to support Hsp70, whereas the cochaperones of Hsp90 include p23, Cdc37, PP5 (protein phosphatase 5) as well as immunophilins: FKBP51 (FK506 binding protein 51), FKBP52 and cyclophilin-40 [17]. Assembly into these complexes is necessary for proper folding of the nascent receptor, enables the hormone-binding process and represses transcriptional activity of unliganded SR [17].

Upon ligand binding the SR becomes increasingly phosphorylated and the Hsp90 complex dissociates [2,17,18]. The process of homo- or heterodimerization then follows, with the latter referring either to the interaction between two different isoforms of the same SR (e.g., PRA-PRB or GRα-GRβ heterodimers) or to one type of receptor binding another as it is for example in case of GR-MR complexes [14,15,19]. Canonically, the dimer initially forms in solution thanks to a contact between the two LBDs and is then further stabilized in the presence of DNA by an interaction of DBDs [2,12,14,15,20,21]. However, the remaining domains may also be necessary, as was shown for the NTD and the hinge region of PR [21], or even sufficient (namely, the hinge region of GR [20]) for a dimerization to occur. SR dimer then undergoes a nuclear translocation, enabled by importin proteins bound to the NLS (nuclear localization signal) located at the junction of the DBD and hinge region [12,15,20,22,23,24].

However, this order of events, where ligand binding precedes dimerization which is then followed by nuclear translocation does not apply in many cases. Hormone binding often occurs in the nucleus where some SRs, like GRα-D isoform, reside constitutively, whereas others, having been recycled after previous transcriptional action, stay ready to rebind ligand [2,14,16].

Classical SR signaling mechanism, also referred to as direct genomic signaling, involves an interaction between SR dimer and a specific DNA sequence known as HRE (hormone response element) which in this case can be also called SRE (steroid response element) [2,24,25]. Mechanistically, the process involves one of the two zinc fingers of the DBD contacting the major groove of the DNA and the other partaking in the aforementioned DNA-dependent dimerization of the DBDs [2,12]. Core motif of an SRE consists of two six-base-pair-long units separated by a three-base-pair spacer. Each of these hexameric half-sides binds one receptor of an SR dimer [2,12]. Based on which SR class they bind, SREs can be described as EREs (estrogen response elements), PREs (progesterone response elements), AREs (androgen response elements) and GREs (glucocorticoid response elements) which serve as binding sites not only for GR but also for MR [15,26]. Multiple chromatin immunoprecipitation and sequencing (ChIP-seq) analyses carried out in order to determine the exact HRE sequences have shown that that the response elements for single SR type appear in many variants and at the same time SREs for different receptors often share the same sequence [27]. For example the GGTACAnnnTGTTCT motif, which was described as the canonical SRE, can serve as a binding site for GR, MR, PR or AR [2,28,29]. Specificity may possibly be achieved via differences in flanking DNA sequences or spacer nucleotides [2,28]. SREs can be found in promoter regions of SR target genes as well as within enhancer sequences located inside introns or intergenic spaces [19,22,24].

Once bound to the SRE, the SR recruits a plethora of coregulator proteins which could be either coactivators or corepressors of transcriptional activity. Both groups perform histone modifications, remodel the local chromatin and interact with transcription machinery proteins, which in case of the former leads to the assembly and subsequent activity of the RNA polymerase II holocomplex, while in case of the latter results in suppression of gene transcription [2,22,30]. Acetylation of the histones at lysine residues by histone acetyltransferases (HATs) is the most commonly described modification leading to gene activation whereas deacetylation, catalyzed by histone deacetylases (HDACs), works in the opposite way [2].

**Figure 3:**
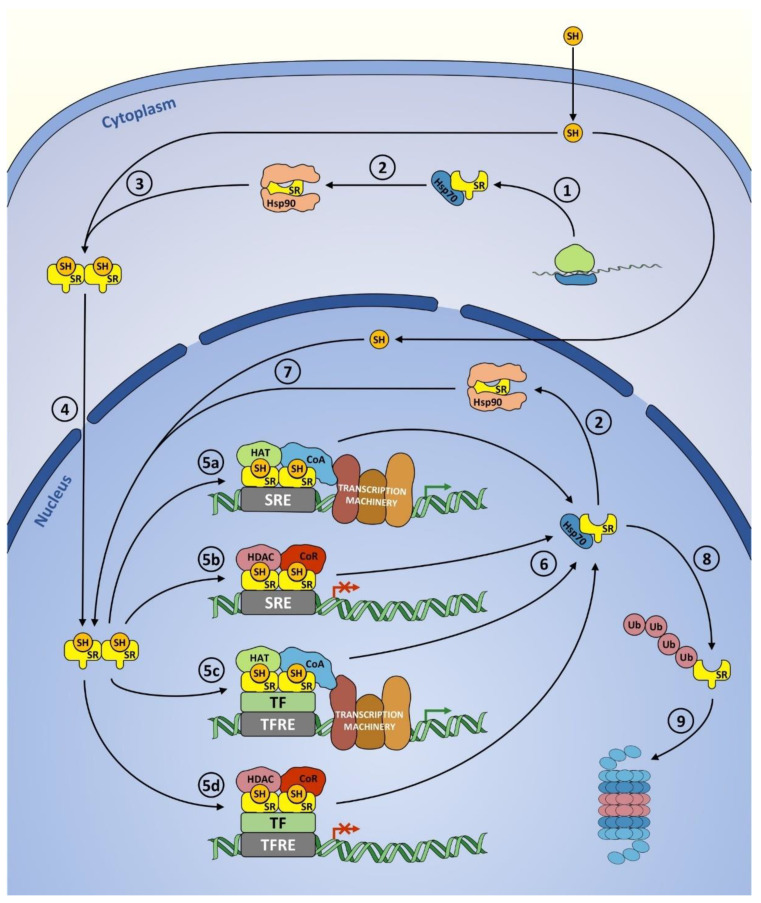
SR synthesis, genomic signaling and degradation. (1) Translation of a SR and binding of Hsp70. (2) Hsp70 to Hsp90 transition. (3) Ligand binding, Hsp90 dissociation and dimerization. (4) Nuclear translocation. (5) Transcriptional action: induction (5a, 5c) or inhibition (5b, 5d) of target gene expression, performed either in the classical mechanism involving SRE-binding (5a, 5b) or by tethering other TFs (5c, 5d). (6) Ligand dissociation followed by disassembly of the transcriptional complex and SR binding to a molecular chaperone. (7) Rebinding of the ligand. (8) Ubiquitination. (9) Proteasomal degradation. SR—steroid receptor, SH—steroid hormone, Hsp 70—heat shock protein 70, Hsp90—heat shock protein 90, SRE—steroid response element, CoA—coactivators, CoR—corepressors, HAT—histone acetyltransferase, HDAC—histone deacetylase, TF—transcription factor, TFRE—transcription factor response element, Ub—ubiquitin. Although HATs and HDACs are classified as coregulators, here they are shown separately in order to emphasize their role. [2,16,17,31,32]. Illustration created using elements from Servier Medical Art https://smart.servier.com/, reproduced under Creative Commons Attribution 3.0 Unported License https://creativecommons.org/licenses/by/3.0/.

Another common mechanism in which SRs regulate gene expression is called indirect genomic signaling and involves SR tethering other DNA-bound TFs, instead of interacting with SRE [2,11,19,24]. Numerous genes, which do not possess SREs in their promoters or enhancers, are regulated by SRs in this manner [11,24]. Both classical and indirect genomic signaling are shown in Figure 3.

Subsequently to hormone dissociation, the SR is detached from the transcriptional complex by molecular chaperones. Hsp90-bound SR then regains the ligand-binding capacity [16,17] (Figure 3). Some SRs can undergo nuclear export which also requires the activity of chaperones [17].

Proteasomal degradation of a SR, following its polyubiquitination, occurs either in the nucleus or in cytoplasm [16,31,33]. Hsp70 is suggested to be responsible for making the triage decision between receptor degradation or stabilization [16]. Normally, Hsp70 recruits Hop to promote transfer of a SR to Hsp90 but in case of a damaged or misfolded receptor, an E3 ubiquitin ligase CHIP (Carboxy Terminus Of Hsp70-Interacting Protein) is recruited instead, resulting in polyubiquitination of a SR [16] (Figure 3).

In addition to regulation of gene transcription, which effects occur typically in minutes to hours after hormone exposure, SRs can also act in so-called rapid or non-genomic signaling mechanism, examples of which, along with other non-canonical modes of SR action, are covered in following paragraphs focusing on each type of SR individually [2,14].

## 3. Estrogen Receptor

Estrogen is an important sex hormone produced predominantly in the ovaries in females and testes in males. Estrogens play an essential role in a number of physiological processes, including regulating energy metabolism, stress responses, mineral balance, as well as sexual development [34,35,36]. Additionally, estrogen is also involved in the function of adipose tissue as well as neuroendocrine, skeletal and cardiovascular systems [37,38].

The biological functions of estrogen are mediated by binding to the ERs: estrogen receptor alpha (ERα) and estrogen receptor beta (ERβ). The genes coding for ERα and ERβ are located on chromosome 6, locus 6q25.1 and chromosome 14, locus 14q23.2, respectively. The ERβ has 530 amino acids and 59 kDa molecular weight, while ERα has 595 amino acids and 66 kDa molecular weight [39]. Five different isoforms of ERα (62 kDa, 53 kDa, 46 kDa, 45 kDa, and 36 kDa) [24], and five ERβ variants (ERβ1–ERβ5) are detected in breast cancer [40].

Estrogen signaling is particularly stimulated or inhibited depending upon an equilibrium between ERα and ERβ activities in the organs. In the 1960s, ERα, the first estrogen receptor, was described [41] and now, the function of ERα is well characterized [42] and ERα is widely used for verifying medication and imaging strategies [43,44]. ERα is predominantly expressed in the uterus and pituitary gland with highest levels in the liver, hypothalamus, bone, mammary gland, cervix, testis, kidney, heart, skeletal muscle, and vagina [45]. In healthy breast glandular epithelium, ERα is expressed by about 30% of the cells [46]. ERα activation stimulates tumorigenesis in various types of cancer, including breast cancer [47].

The second one, ERβ, has unique functions and shows potential as a novel target for pharmacological intervention [48,49]. The common expression of ERβ is detected in luminal and myoepithelial cells in the normal breast but also in subcutaneous adipose tissue [50] and testis, prostate, ovary, uterus, and brain tissues [51]. Changes in estrogenic signaling pathways have been discoursed in the physiological and pathological processes [52], neuronal-mediated contractions of the gastrointestinal tract [53], recovery of reproductive system injury [54], anxiolytic effects [55], and diseases such as Parkinson’s disease [56] or endometriosis [57]. Furthermore, ERβ has been shown to take part in the pathological process of various cancers, e.g., colorectal cancer [58], prostate cancer [59] or duct carcinoma [60]. ERβ expression is also found exclusively in the granulosa cells [61,62]. Both ERα and ERβ1 require ligand binding for ER target gene transcription [63]. Analysis of ERα and ERβ tissue distribution suggests that ERs have high specificity on the target tissue [64].

In ligand-dependent ER signaling mechanisms, the binding of estrogen with ER causes a conformational change, which allows various coregulators to stimulate transcription of ER-target genes. As it is for other SRs, the ligand/estrogen-dependent mechanism is further classified into direct genomic or classical, indirect genomic or non-classical, and non-genomic mechanisms of action [45,65,66].

Helix 12 is the functional core of AF2 and is very conserved in ligand-binding domains. The process of binding to a ligand can alter the configuration of helix 12, which leads to an agonistic or antagonistic form of transcriptional regulation [67]. Attachment between ER and hormone results in a change of conformation in the ligand-binding domain that allows helix 12 to interact with coactivators. The resulting genomic reaction necessitates coactivator binding and is proportional to the magnitude of the reaction. Alternatively, ERα binds to the DNA with inactive status in the absence of hormones [68,69].

Nuclear factors such as pro-factor FOXA1 (Forkhead Box A1) may affect direct DNA binding by recruiting chromatin at the binding site to remodel it. The chromatin is opened, allowing the ER to enter its regulatory DNA site [70].

In addition to its ability to directly regulate gene expression, estrogen also affects cell signaling and cellular function through rapid membrane-initiation events. Many signaling processes rely on estrogen receptors localized to the plasma membrane. Lipid rafts are critical for ER plasma-membrane localization and play a key role in its membrane-priming effect [71]. Together, the integration of these cellular signaling pathways can mediate genomic activities and rapid nongenomic effects independently and/or complementarily, which activates the effects of estrogen through hormonal response.

The last proposed mechanism of the operation of the ER is opportunity-based. ER can regulate cellular responses without hormones by being activated by components of growth factor signaling pathways, which is attributed to the phosphorylation process of some serine residues on the ER [72].

In premenopausal women, estrogens are predominantly produced by the ovary [73]. The hypothalamus releases gonadotropin-releasing hormone (GnRH), which stimulates the secretion of follicle-stimulating hormone (FSH) and luteinizing hormone (LH). FSH stimulates the biosynthesis of estrogens in growing ovarian follicles, which then act on the hypothalamus to induce the production of LH. An acute rise in LH triggers ovulation and the development of the corpus luteum. After menopause, the ovaries produce negligible levels of estrogens [74]. The importance of gonadal steroidogenesis in normal breast development and in the origin of breast cancer is emphasized by the fact that early menstruation and late menopause are linked to a higher risk of breast cancer [75]. Similarly, late menarche and early menopause (before the age of 40) result in a significant reduction in the risk of developing breast cancer [76,77]. It is somewhat paradoxical, therefore, that the majority of breast cancers occur after menopause, when circulating estrogen levels are low.

ERβ plays an important role in the cell actions and extracellular matrix (ECM) composition of breast cancer cells and may have an effect on important chemokine receptors [78,79] as well involved in the beclin1-dependent autophagic cascade [80].

## 4. Progesterone Receptor

Another receptor, that plays a crucial role in both normal and cancerous mammary gland cells, is the progesterone receptor (PR) which is encoded by the PGR gene found at the 11q22.1 locus (HUGO Gene Nomenclature Committee https://www.genenames.org/ [81]). There are two main isoforms of PR: PRA and PRB, both being a product of the PGR gene, but transcribed using alternative promoters, which results in PRA lacking 164 N-terminal amino acids [82].

### 4.1. Mechanism of Action

In the nucleus PR binds to promoter or, more commonly, enhancer regions of numerous genes [19]. ChIP-seq analysis of T47D human breast cancer cells performed by Ballaré et al. revealed more than 25000 PR-binding sites in the DNA on the basis of which a PRE sequence was identified [83]. Nevertheless, several millions of human DNA sequences have been then shown to match the proposed PRE, raising the question of what factors may determine whether a certain site would be occupied by the PR or not [83]. One possible explanation, though probably insufficient to account for such a vast disproportion, is the involvement of other TFs including such acting as so called pioneer factors—Proteins responsible for chromatin remodeling in order to provide access to the DNA for other TFs [19,83,84,85]. Indeed, two members of the STAT family of transcription factors (STAT3, STAT5A) as well as FOXA1 have been postulated to play such role towards the PR [84,85]. However, PR binding does not seem to be dependent on the dislocation of nucleosome core proteins by pioneer factors which is necessary in case of many other TFs. It is suggested that FOXA1 factors occupying the PR-binding sites act on chromatin rather by displacing the H1 histone alone instead of remodeling the whole nucleosome [29,83]. Interestingly, PR itself can act as a pioneer factor for other TFs [84].

Another ChIP-seq study focused on distinguishing between the cistromes of two PR isoforms, showing that the overlap between them is lower than one might expect despite similar binding sequences [82]. Likewise, expression analyses indicate that transcriptomes of PRA and PRB differ significantly, highlighting the need for an isoform-oriented approach in designing future PR-targeting therapies [82,86,87,88]. However, the difficulty in deeper understanding the PR isoform-specific actions lies in the fact that in vivo most cells coexpress both PR isoforms resulting in coexistence of three different dimer variants, namely the PRA and PRB homodimers as well as the PRA-PRB heterodimer, with most likely distinct cistromes and transcriptomes [30,86]. While most studies investigating the PRA- and PRB-dependent gene expression patterns were based on cell lines modified to synthesize only one isoform, providing insight to the genomic action of the homodimer forms alone, a research by Khan et al. compared gene expression in PRA(+)PRB(−), PRA(−)PRB(+) and PRA(+)PRB(+) breast cancer cells showing that indeed a certain subset of genes is regulated exclusively by the PRA-PRB heterodimer [87].

PR is capable of exhibiting both activatory and repressive effect on gene transcription. Several factors have been proved to determine whether the expression of a given gene would be promoted or inhibited by the PR. The localization of the PRE in the enhancer sequence of a target gene for instance, is more associated with transcriptional activation, whereas genes harboring a PRE within their promoter region are more commonly repressed upon PR binding [84]. Furthermore, isoform-specificity of PR action is reflected also here, as studies indicate that in most cellular contexts PRB can be classified as an activator, and PRA as a repressor of transcriptional activity [89]. The reason for this lies in the fact that there are many differences between coregulator interaction profiles of PRA and PRB among which a notable one is that PRA is unable to efficiently recruit coactivator proteins NCOA1 and NCOA2, while showing stronger affinity to the corepressor NCOR2 (SMRT) than PRB (Table 1) [30,82,90]. The genomic outcome of PR action depends also on local coregulator expression which has been proved to be different in distinct tissues. It also seems to be affected by the phase of the menstrual cycle or the occurrence of cancerogenesis [30].

PR indirect genomic signaling involves tethering such TFs as AP-1, SP1 or STAT3, whereas the rapid, extranuclear effects exhibited by the receptor include modulation of certain cellular pathways via direct interactions with cytoplasmic or membrane-associated proteins [11]. Furthermore, under certain conditions, PR can function independently of ligand binding or without dimerization [92,93].

### 4.2. Post-Translational Modifications

Post-translational modifications play a huge role in regulation of PR activity, affecting for example its stability, subcellular localization or promoter selectivity [94,95]. They include phosphorylation, ubiquitination, SUMOylation, acetylation and methylation [19].

Several protein kinases are able to catalyze the phosphorylation of the PR, including DNA-PK (DNA-Dependent Protein Kinase), PKA (Protein Kinase A) as well as mitogenic kinases such as CDK2 (Cyclin Dependent Kinase 2), CK2 (Casein Kinase 2) or members of the MAPK (Mitogen-activated Protein Kinase) family (e.g., MAPK1 (p42 MAPK) or MAPK3 (p44 MAPK)) [19,95,96]. Phosphorylation partakes in a classical, hormone-induced PR signaling pathway. Ser102, 162, 294, and 345 are involved in this process [19]. However, intensive PR phosphorylation can also lead to a ligand-independent nuclear translocation which often takes place in case of breast cancer due to excessive activity of mitogenic kinases [95]. Phosphorylation of Ser294 by MAPK or CDK2 and Ser400 by CDK2 have been shown to work in such mechanism. In case of the former, the receptor becomes sensitized to lower hormone concentrations. Moreover, the ligand binding process occurs when the PR is already inside the nucleus which significantly accelerates the occurrence of transcriptional effects. Ser400 phosphorylation, on the other hand, enhances the transcriptional activity of unliganded PR [92,94]. Phosphorylation pattern is also an important determinant of PR promoter selectivity. Namely, Ser81 phosphorylation by CK2 results in increased expression of STAT5A and WNT1 and thus plays a role in breast cancer cell biology also possibly affecting the process of inflammation related to breast cancer development and progression [95]. Notably, Ser81 is not present in the truncated PRA isoform [94]. 

E3 ubiquitin ligases RPF1 (Ribosome Production Factor 1 Homolog), E6-AP as well as BRCA1 can be associated with polyubiquitination of PR. This modification marks it for proteasomal degradation which, paradoxically, leads to increased transcriptional activity, as different stages of transcription may require the receptor either present or removed from its DNA binding site. Indeed, increased ligand stimulated activity of many nuclear receptors is tightly linked with their augmented proteasomal degradation [19,94].

SUMOylation is a post-translational modification involving the attachment of a small protein called SUMO (Small Ubiquitin-Related Modifier) to a target protein. The process resembles very much of ubiquitination with its final ligation step also being catalyzed by enzymes called E3 ligases. However, unlike ubiquitination, SUMOylation does not target proteins for proteasomal degradation but rather stabilizes them or modifies their function. PR can be SUMOylated at its Lys388 residue by an E3 SUMO ligase PIAS1 (Protein Inhibitor of Activated STAT 1) which results mostly in a decreased hormone-dependent stimulation of gene transcription, probably due to promotion of interaction with corepressors. Such mechanism of SUMOylation-induced modification of transcriptional activity have been demonstrated in case of other TFs, like PPARγ (Peroxisome Proliferator Activated Receptor γ). PIAS3 has been also proved to affect PR function but it is not clear whether this interaction involves its E3 SUMO ligase activity. Removal of SUMO from the PR, catalyzed by SENPs (SUMO/Sentrin-Specific Proteases), results in upregulated transcription of target genes. SUMOylation-mediated regulation of PR target genes transcription is much more pronounced regarding genes possessing multiple PREs rather than just a single one [94]. Gene signature associated with increased activity of deSUMOylated PR corresponds with endocrine resistance and poor outcome in breast cancer [19].

Acetylation of the PR can occur on a lysine (Lys; K) residue within a conserve KXKK motif located in the hinge region, or on Lys183. In response to hormone stimulation, the KXKK acetylation facilitates the nuclear translocation thus accelerating PR-stimulated transcription of such genes as MYC [19,23]. Lys183 acetylation, which can be catalyzed by p300 coactivator, also enhances PR transcriptional activity, but it does so by increasing its DNA-binding capacity [19].

Last reported post-translational modification of PR is the Lys464 monomethylation which most likely suppresses its transcriptional activity [19,97].

### 4.3. Role in Healthy Breast

In healthy mammary gland, the main effect of progesterone stimulation is cell proliferation and differentiation leading to breast development and growth—The process which takes place mostly during puberty and lactation [19,92]. This mitogenic function of the PR is exhibited by transcriptional upregulation of cell division associated genes, such as CCND1 (Cyclin D1), MYC or FOS and JUN (components of AP-1 transcription factor) (The Signaling Pathways Project database https://www.signalingpathways.org/index.jsf) [11,98,99,100]. However, most of the cells aren’t affected by progesterone directly, as studies on mouse models indicate that only about 20–40% of breast luminal epithelial cells express the PR [11,19]. Therefore, the progesterone-induced proliferation occurs in two phases. First, during initial 24 h after progesterone exposure, the PR-positive cells proliferate and synthesize paracrine mitogenic factors, the most important of which is TNFSF11 (Tumor Necrosis Factor Superfamily Member 11), also commonly known as RANKL (Receptor Activator Of Nuclear Factor Kappa B Ligand); which then trigger the proliferation of remaining cells [11]. TNFSF11 is a direct PR target gene. It exhibits its activity by binding to a TNFRSF11A (TNF Receptor Superfamily Member 11a; RANK) membrane receptor which then activates the mitogenic NF-κB (Nuclear Factor Kappa B) signaling pathway [11]. Other paracrine mediators induced by PR in mouse mammary gland include amphiregulin (AREG) and WNT4 (ligands of EGFR (Epidermal Growth Factor Receptor) and FZD (Frizzled) class receptors, respectively), which also act as proliferative factors and trigger the formation of new ducts and secretory alveoli; as well as calcitonin, with its receptors (CALCR) located in breast exclusively on myoepithelial cells, and a chemokine CXCL12 responsible for stimulation of progenitor cells via the CXCR4 receptor [11]. The similar stem cell regulatory function is exhibited by the Notch signaling pathway, with Notch receptors ligands (i.e., JAG1 (Jagged Canonical Notch Ligand 1), DLK1, DLK3 (Delta Like Non-canonical Notch Ligand 1 and 3)) also being upregulated by the PR [101]. Interestingly, it has been suggested that growth hormone (GH) is secreted locally in the breast where it acts as a paracrine mediator and that its synthesis is also stimulated by progestins [101].

### 4.4. Role in Breast Cancer

Progestin-induced PR activity is a well-known factor driving carcinogenesis in the breast. Large epidemiological surveys have shown that usage of progestins as components of either contraceptives or hormone replacement therapies markedly increases breast cancer risk [11,102,103,104,105,106,107]. On the other hand, once the tumor is established, loss of PR expression in later stages of its development is associated with less differentiated and more aggressive phenotype accounting for worse prognosis [19,92].

Mouse model studies indicated a crucial role of PR action in initiation of tumorigenesis. It is the paracrine signaling, mediated by PR-induced TNFSF11 as well as, possibly, WNT1 and WNT4, that contributes majorly to this process, however, its role gradually decreases as the tumor enters later stages of development. Moreover, there is an observed increase in relative abundance of PR-positive cells from aforementioned 20–40% in normal breast to approximately 50% in invasive cancers, which suggests a shift from paracrine to autocrine mode of signaling as a main driver of tumor progression [11,92]. The main mediator of this autocrine signaling is most likely WNT1 [108]. Interestingly, the classical WNT/ꞵ-catenin pathway doesn’t seem to play a major role in this process. It is rather postulated that, upon WNT1 binding, FZD class receptors, as members of the G-protein coupled receptor (GPCR) family, activate the Ras/MAPK cascade by rapidly stimulating, in a still not fully elucidated mechanism, membrane-anchored matrix metalloproteinases (MMPs), e.g., members of the ADAM (A Disintegrin And Metalloproteinase) family, which then cleave away membrane-bound EGFR ligands, such as HBEGF (Heparin Binding EGF Like Growth Factor), TGFA (Transforming Growth Factor Alpha) or AREG, allowing them to interact with their receptors [108,109,110]. Alternatively, GPCRs can possibly induce the Ras/MAPK pathway without the involvement of MMPs. SRC non-receptor protein tyrosine kinase (PTK) is likely to be involved in both of these mechanisms, as, subsequently to being activated by Gꞵγ subunit or, supposedly, DVL (Dishevelled) in case of FZD signaling, it may either partake in MMP stimulation or directly phosphorylate cytosolic domain of EGFR to initiate the mitogenic cascade [110,111,112,113]. Overall, this PR-induced autocrine signaling accounts for very potent stimulation of cell proliferation, as it further augments the already strong pro-proliferative action of PR itself as a TF. Indeed, cyclin D1 has been shown to be upregulated upon progestin treatment both directly by PR-dependent transcription and indirectly via the autocrine mechanism [108].

There are further examples of crosstalk between PR and MAPK signaling. SRC kinase, additionally to the aforementioned mechanism, can be activated by a direct interaction with the PR which serves as an example of PR non-genomic effects. This occurs through the SH3 (SRC Homology 3) domain of SRC binding the proline-rich motif located within the NTD of the PR [19,92]. Moreover, the PR-MAPK interplay occurs in both directions, as PR activity is substantially potentiated upon the said phosphorylation of Ser294 by MAPKs. This process drives the progression of breast cancer by reducing its dependence on progestins, thereby contributing to the development of resistance to hormone therapy [92].

PR also interacts extensively with STAT transcription factors which play an important role in breast physiology, as prolactin (PRL) signaling occurs via the PRLR/JAK2/STAT pathway (PRLR—Prolactin Receptor, JAK2—Janus Kinase 2) [19]. Transcription of STAT5A, the main mediator of PRL impact on gene expression, is induced by the PR [19,98,99]. As mentioned above, this effect depends on Ser81 phosphorylation which, interestingly, also promotes interaction between PR and STAT5A protein to stimulate the expression of other genes, such as WNT1 and, possibly, TNFSF11 [11,19,114]. Notably, Ser81 phosphorylation by CK2 requires DUSP6 (Dual Specificity Phosphatase 6) acting as a scaffold to bring PR and the kinase close together [95]. Likewise, PR cooperates also with STAT3. They have been shown to associate at promoter regions of such genes as BCL2L1 (Bcl-X) or CDKN1A [19,99].

PR also promotes invasiveness of breast cancer by downregulating GATA3. This TF functions in the mammary gland to promote cell fate determination and maintenance thus, in case of breast cancer, it prevents the loss of tumor differentiation and subsequent metastases. Izzo et al. have shown that both PR isoforms are able to repress the transcription of GATA3 and that this process involves the recruitment of EZH2 corepressor [11,115].

An extensive crosstalk between PR and other SRs is observed in breast cancer cells. PR and ER have been shown to associate in cytoplasm as well as at multiple DNA binding sites, including both progesterone and estrogen response elements. PR-induced transcription of such genes as CCND1 and MYC requires ER colocalizing with PR at the gene promoter [116]. Importantly, studies show that cotreatment with estrogen and progestin redirects ER chromatin binding profile towards sites corresponding to the PR cistrome, and, consistently, results in a gene expression signature similar to that observed after the exposure to progestin alone, but not estrogen alone, thus indicating that PREs are more commonly occupied by ER-PR complexes than EREs [117,118]. Consequently, this progestin-induced reprogramming of estrogen signaling suppresses the expression of ER target oncogenes. In line with that, progestin treatment was shown to inhibit the estrogen-driven growth of breast cancer xenografts, acting synergistically with an ER antagonist [117]. However, antiprogestins were found to induce the regression of breast tumors more potently than progestins, which remains consistent with the known mitogenic effect of PR action [82,118]. Furthermore, PR antagonists retain the ability to affect estrogen signaling but act most likely by repressing the ER transcriptional activity rather than reprogramming it [82,119].

In accordance with the aforementioned discrepancies in cistromes and transcriptomes of the two PR isoforms, PRA and PRB have been shown to differentially affect the breast cancer biology, including the estrogen signaling. Namely, both subtypes redirect ER towards new chromatin binding sites, however, much fewer of those is observed in case of PRA which can be, therefore, treated as a repressor of estrogen signaling [82]. In normal tissue the PR isoforms are present in almost equal amounts but in breast cancer cells the PRA:PRB ratio is often disrupted [11,120,121]. PRA predominance occurs more frequently which may seem unexpected, as PR mitogenic activity involves mainly transcriptional induction, whereas PRA is more commonly associated with repressive function. However, McFall et al. indicated that PRA could promote the invasiveness of breast cancer in a mechanism involving a certain aspect of ER-PR corsstalk. Namely, PRA could function as a transdominant repressor of ER to inhibit ER-mediated regulation of miR-92a-3p and miR-26b-5p transcription, or directly affect the expression of these miRNA molecules, ultimately leading to reduction of the invasiveness-suppressive effect exhibited by estrogen in later phases of tumor development [89]. Another approach to explaining the phenomenon of increased PRA:PRB ratio states that it may be in fact the PRB isoform that is more active which at the same time could be the very reason for its decreased cellular concentration because elevated transcriptional activity is associated with augmented proteasomal degradation [11,94]. To this day it has not been fully solved which isoform actually exhibits worse tumorigenic properties. In breast cancer xenografts PRB predominance was shown to account for faster tumor growth and reduced tamoxifen responsiveness [122]. On the contrary, other studies found that patients with PRA-rich breast lesions were more likely to relapse after treatment that included tamoxifen [123,124]. Two more recent studies comparing clinical outcomes in breast cancer patients with gene expression signatures indicative of PRA or PRB predominance yielded inconsistent results, one showing shorter relapse-free and metastasis-free survival [125] and the other better overall survival [82] in patients with PRB-rich tumors. However, what seems clear is that PRA predominance predicts better antiprogestin responsiveness [125,126]. Indeed, recently published first results from the MIPRA study, investigating the use of PR antagonist mifepristone for the therapy of breast cancer with high PRA/PRB ratio, seem to be promising [7].

## 5. Androgen Receptor

### 5.1. Metabolism of Androgens in Females

The androgen synthesis in women occurs mainly in the adrenal glands (25–50%, depending on hormone, with DHEA-S created only therein), in the ovarian stroma (25–50% respectively) and the ovarian theca (20% of DHEA). A notable feature of the women’s androgen synthesis pathway is the fact that DHEA-S and testosterone-preceding androgens stem in significant amounts from peripheral conversion of estrogens [127].

### 5.2. Androgen Receptor Structure and Signaling Pathways

The androgen receptor (AR) in human is a 110-kDa protein with its gene located on the X chromosome, in the locus Xq11–Xq12 [12]. Its natural ligands include dihydrotestosterone (DHT), testosterone (with a much lesser potency) and other androgen pathway metabolites that are present in very small concentrations in blood [6]. When unactivated, AR resides in the cytoplasm, bound to heat shock proteins (Hsp90, Hsp70, Hsp56, Hsp27). Contrary to other steroid receptors, its main transcriptional activity is mediated by AF-1 (AF-2 in the others) [128].

Although expressed predominantly by male genital organs, it is also present at variable levels in the cervix and vagina and the acini and ducts of the breast [129]. Approximately 20% of breast epithelial cells express AR in the healthy tissue [130]. AR is responsible for growth and differentiation in normal breast. Its knock-out or blockage in mice led to ‘reduced ductal branching, decreased lobuloalveolar development, fewer milk-producing alveoli (...), altered mammary gland development/morphology’. AR activity is corollary to proliferative stimuli of ERα and serves to counteract and regulate excessive proliferation of breast tissue, that interplay leads to normal ductal development of the breast. In fact, in ERα- cell lines like MDA-MB-453, more than half incidents of AR-DNA interaction occurred at ER-dependent genes; this action seems to be mediated by FOXA1 [131]. Some reported cooperation of AR and ER in growth induction, although it is related more to the binding of the other receptor’s ligands than to cooperation [132]. Other studies show that in certain cancer lines (e.g., MCF-7) and in certain ERα-negative breast cancers (the molecular apocrine subtype) AR can successfully mimic the cistrome of ER and be pro-proliferative, as it is antiproliferative in luminal breast cancers. AR supports HER2 expression by activating WNT and MYC [133]. In a study, ER- breast tumors exhibited cross-talk between AR and HER2, resulting in their proliferation [134].

The first exon of the AR gene, coding the terminal peptide, exhibits a polymorphism of CAG and GGN (polyglutamine and polyglycine stretches, respectively). Excess repetition of CAG may lead to decreased transactivation and transcription of the AR (with greater binding affinity to DHT). It is speculated that shorter CAG polymorphism increases the risk of prostate cancer (although the relation with severity and age of onset is unclear). Shorter CAG stretches are more frequent in African-Americans [135]. One of the probable mechanisms of diseases caused by elongated CAG repeats includes the fact that they destabilize normal protein conformation, leading to formation of antiparallel β-sheet, aggregates and fibrils similar to those seen in prion and neurodegenerative (e.g., Huntington’s disease) diseases [136]. The lesser-studied GGN repeats also influence transactivation of the AR, however, in this case the 23-repeat-sequence is considered the peak of maximal transactivation and the most optimal for AR function, as the lower and higher amounts are associated with diseases (non-neoplasmal) [137]. The meta-analyses on the effect of GGN repeats on prostate carcinogenesis have been inconclusive, with some proving the relation of shorter GGN sequences and prostate cancer [138] and some finding no significance thereof [139], contrary to the clearly proven positive correlation of prostate cancer and CAG repeats [139]. Some studies show that longer CAG repeats are related to earlier diagnosis of breast cancer in women with present BRCA1 mutations [140], although it is contested by others [141] on various bases, including indefinite threshold between ‘long’ and ‘short’ CAG repeats, populational differences and the heterogenic cell strains in tumor [142].

BRCA1 induces activity of AR by binding to the activation function domain of NTD. In BRCA-deficient heterozygotes, longer CAG trinucleotide repeats in AR NTD have shown to cause higher risk of BRCA-double null breast cancer—however, it was refuted by other studies. A specific PIK3CA mutation in kinase domain causes higher expression of AR in cancer cells, both in cells with full SR expression and ER, PR deficient [132].

AR forms with constitutive activity are known for prostate cancer; for example, the AR-Vs (variants) are located mainly in the nucleus (also in breast cancer lines like MDA-MB453) and present constant activity. Lacking AF-2 and a part of LBD, their activity is performed by AF-1 and can be compared to activity of normal ARs [128,133]. A wide range of these C-terminally truncated forms is known (AR-V1 to AR-V18, AR45, ARQ640X, trAR, exon-skipping forms, e.g., ARv5es, ARv56es, ARv567es=AR-V12, ARv7es) [128,133,143]. These forms of AR are responsible for antiandrogen resistance in castration-resistant prostate cancer [143]. Normal AR receptors dominate in luminal A cancers and are expressed in lowest amounts in basal cancers [133]. AR-V7 presence is very common (51.5%) in breast tumor samples, especially in ERα- cancers and is correlated with higher levels of HER-2, other common occurrences of mutated AR include AR45 (25.0%), AR-V1, AR-V2 or AR-V4 (9.3%), AR-V3 (16.3%), AR-V9 (13.7%) and AR-V13, AR-V15 or AR-V18 (19.5%). Many breast cancer cell lines, including MDA-MB-453, T47D, ZR-75-1 and MCF-7, express AR-V7 [133].

AR is known to possess sixteen phosphorylation sites, although some are occupied only in cancer cell lines or after medication (e.g., antiandrogens). Several of them are related to oncotic processes of prostate cancer (Table 2) [128].

AR can be acetylated in the hinge region (K630, K632, K633), in the two latter by p300 and p/CAF, or by Tip60 and deacetylated by HDAC1 and SIRT1. This modification leads to increased transcriptional activity of the receptor and was shown to occur in several prostate cancer cell lines; comparably, deacetylation leads to decreased AR activity [128]. The mentioned lysines (K630, K632) can also be methylated by Set9 and it leads to different expression profiles (expression of PSA, suppression of KLK2, TMPRESS2, NKX3.1, NDRG1). AR can be demethylated by lysine demethylase family (KDM) proteins: KDM4A, KDM4D, KDM4C, KDM4B [128]. K845 and K847 residues can be ubiquitinated: RNF6 causes monoubiquitination and increases transcriptional activity by polyubiquitination, while E3 ligases MDM2 and CHIP cause only polyubiquitination and consequent proteasome degradation. ZIPK coactivator causes polyubiquitinylation and increased activity of AR. E3 ligases such as PIAS1 and PIASxα cause SUMOylation of AR in the cytoplasm, while their E1 and E2 counterparts seem to serve the same function in the nucleus. K386 and K520 lysines were found to undergo SUMOylation, which in case of AR leads to decreased transcriptional activity, however, several other putative SUMOylation locations are known and their effects vary between cell lines, agent proteins, activation state or treatment. Surprisingly, SUMOylation site mutation caused lower half-life of unbound AR and prolonged half-life in bound AR [128].

AR coregulators encompass numerous proteins, with several of particular interest in cancer (Table 3) [162,163].

It is estimated that androgen receptors are present in about 80% of invasive breast cancer cells, with the highest occurrence (95%) in ER-positive, and the lowest (10–35%) in triple-negative breast cancers (TNBC) [132,165]. In fact, AR is the most commonly expressed nuclear receptor in breast cancer overall and is overexpressed in 62% of breast tumor samples [132]. Other studies have reported highest presence of AR in luminal A cancers and lowest in TNBC [131]. ARs are present in 25% of metastases [131] and in majority of ER-, HER2+ apocrine tumors [132]. In several cases (25%), AR is the only sex hormone receptor expressed by distant metastases [132]. The absence of androgen receptor correlates positively with mean tumor size (in ER+ tumors), Nottingham grade and the presence of necrosis [165], AR-positivity can be associated with older age of onset, lower stage and grade of cancer, 27% reduction of overall breast cancer mortality, 46% reduction of mortality for ER+ breast cancers and 62% increase of mortality for ER- breast cancers, although only for the 7 years postdiagnosis, as a 48% increase in mortality in AR+ cancers was noted thereafter. The increasing degree of AR-positivity in ER- cancers is related to poorer prognosis [166]. In ERα+ cancers AR is positively related to lower grade, reduced node involvement, longer disease-free survival and these relations are AR level-dependent [131].

AR expression reduces with tumor grade progression in both ductal carcinoma in situ (DCIS) and metastatic carcinoma, yet is expressed at a higher rate than ER at any tumor grade [132].

Possible causes for ER and AR crosstalk include AR coexpression with ER, mutual stimulation by respective steroids, inhibitory binding of testosterone by ERα and correlated upregulation of ERβ [142]. AR upon activation can bind by the DBD to EREs (e.g., vitellogenin, CTSD, PGR) and block ERα transactivation and 17β-estradiol growth stimulation in MDA-MB-231 cells. It is disputed that this activity is responsible for effectiveness of aromatase inhibitors in breast cancer therapy or androgen therapy with promising results [167]. The androgen pathway may increase endocrine therapy (tamoxifen, anastrozole, fulvestrant) resistance in ER+ cancers by cross-talk with estrogen pathway. AR can stimulate HER2 pathway through phosphorylation of ErbB2, in turn, the AR’s expression is prompted by PI3K and mTOR, descendants of HER2 [142,168].

The investigation of AR influence on TNBC yielded mixed results, ranging from increased mortality and metastases through no relation to better prognosis and lesser node involvement [131].

Luminal AR TNBC subtype is known to present ERα-similar effects under AR stimulation. Both AR and ERα can form ternary complexes with SRC and MNAR (PELP1), activating various pathways downstream, including MAPK. Direct corepression is also possible, either with AR NTD binding ERα LBD in presence of estradiol or by AR futile interaction with EREs (mediated by AR LBD) [131] One possible mechanism of AR mimicking ER is its influence on p21—In absence of EGFR or other MAPK pathway stimulation, AR activation leads to proliferation (in presence of the forementioned, AR activation is suppressive).

AR expression is positively correlated with HER2 expression; it was shown that AR presence has beneficial effects only in tumors expressing HER, with no influence on outcome of HER2- luminal B cancers [131]. Hyperactivation of HER2 leads to overactivation of AR and in turn causes increased transcription of HER2 gene. PSA expression is stimulated by AR and was shown to indicate more benign forms of breast cancer, lower histological grade and ER positivity, other studies report on the contrary [131,132]. PSA level can be assessed from patient serum or nipple aspirate fluid [132].

Androgen receptor therapy considered in breast cancer includes two main strategies: agonist administration in ERα+ cancers and antagonist administration in AR+ TNBC (the ‘molecular apocrine disease’), in this case the evaluated drugs include bicalutamide, enzalutamide, abiraterone acetate and orteronel [131]. Archaic therapies featuring sole testosterone administration fell into disuse in about 1970 due to discovery of androgen to estrogen conversion in vivo, lower effectivity than estrogen-related therapy and introduction of SERMs like tamoxifen. Combinatorial therapy targeting MAPK pathway and AR was shown to reduce tumor cell viability and tumor burden. AR expression is a positive outcome factor in tumors treated with medroxyprogesterone acetate [132]. Inhibition of SRF, a transcription factor related to AR in triple negative cancer lines (MDA-MB-231 and HS578t) by CCG1423 (N-[2-[(4-Chlorophenyl)amino]-1-methyl-2-oxoethoxy]-3,5-bis(trifluoromethyl) benzamide) reduced cell viability and migration [164]. Surprisingly, AR+ cancer cell lines reduce their proliferation in response to either AR agonists or AR antagonists, in the latter case even AR- lines (BT-20, MDA-MB-468, SUM-159PT) are susceptible to enzalutamide and bicalutamide (this can be explained by the drugs’ affinity towards GABA-A, CYP27A1 and PR). Treatment of TNBCs with AR agonists, especially accompanied by VDR agonists, leads to G1 phase arrest, increase of apoptosis, reduction of tumorsphere formation efficiency and cancer stem cell features (by deactivation of CD49f, SOX2, and Notch pathways), with epithelial transformation (increase of claudin-4, cytokeratin 18, down-regulation of cytokeratin 5 and vimentin) [134].

## 6. Glucocorticoid Receptor

The glucocorticoid receptor’s gene is located in the long arm of chromosome 5 (5q31Y32) and alternatively called NR3C1. When inactive, GR receptor is bound to several chaperone proteins, including hsp90 (by LBD) [169], hsp70, p23 and FK506-family immunophilins [14]. Hsp90 with p23 and Hsp70 maintains structural and functional integrity of LBD, promoting nuclear localization of active GR and influences the transcription of GRE-regulated genes. Hsp90 is overexpressed in some cancer cells, leading to abnormal activity of GR, caused by excess affinity of LBD for ligands, aberrant colocalization in the nucleus and increased GR transactivation [169]. GRα is known to heterodimerize with GRβ, AR and MR [170]. GR can bind not only GRE, but also AP-1, AML1, UNKN, NF-kB, HNF3, TAL1, and NF1 response elements [26].

Due to its immense activity, the coactivators and corepressors of GR are numerous, several are described in Table 4.

At least seven sites of GR phosphorylation are known [171], but it is unclear what enzymes cause this modification in vivo (in vitro proven for CDKs, MAPKs, JNKs, GSK-3). As with other receptors, phosphorylation leads to increased interaction with cofactors, longer half-life, nuclear translocation, alternation of GRE-dependent transcription and increased glucocorticoid-induced apoptosis. Ubiquitylation of Lys419 causes the removal of GR from nucleus and degradation, but unspecified ubiquitylations of GR also resulted in its longer attachment to DNA and altered transcription. SUMOylation (lysines 277, 293, 703) presents effects that are strongly dependent on a given cell type, can lead to repression of AP-1 and NF-κB sites, inflammatory proteins and to increased affinity towards NCOR1 and SMRT, but in general seems to increase expression of genes related to cell proliferation, growth and survival. GR can be acetylated at Lys494 and Lys495 by CLOCK and BMAL1. That leads to reduction of its effects on the cell; this modification can be reverted by HDAC2. Nitrosylation of GR may occur at four cysteine residues, however, its consequences are unknown, inferring from other receptors, nitric oxide reduces their affinity to DNA and dimerization rate [174].

Several isoforms of GR have been described to this day: GRα (the ‘standard’ form), GRβ—Which is present in the nucleus (however, some studies revealed its significant presence only in the cytoplasm [175]), has no LBD [169], exhibits constant activity and suppresses the effects of GRα. Apart from that, GRβ possesses other specific DNA targets and is responsible for glucocorticoid resistance. GRγ is similar to GRα but exerting less potent effect, GRδ—Expressed in some tissues, GR-P, found in some cancers and other forms, scarcely described (GR-A, GR-DL1, GR-NS1, GR-S1). Multiple variations, caused by alternative translation start (GR-A to GR-D) [14], are tissue-specific and contribute to differentiated effects of glucocorticoids on specific organs due to varied transcription profile and coactivators [171]. Only about 10% of their targets are shared between them, the D forms reside exclusively in the nucleus and are the most resistant to glucocorticoid apoptosis induction (and less active in general), in comparison, the C forms are the most prone thereto [14]. Breast cancers do harbor different GR alternative translation start isoforms (GRα A-D) [176].

About 299 genes were shown to be regulated by the ‘defective’ GRβ (in HeLa cells), its activation upregulated S100P, ECM1, CGA, FOLR3, ADSSL1, MAFG, ENPEP, SLPI, CACHD1, TRIM29, LOC389203, FGF12 and downregulated FBLN2, UBE2U, SRPK2, CDH2, SELS, VIM, PTPRJ, C8orf4, LIMA1, TCTN1, ABCB1, TGFB2, LUM, TPM1, LOC729113, CA5A, LAMA4, STX1B, UGCG and TNC. For GRα, these were SOX2, CANT1, NEK2, ACOX1, TWSG1, ABCG8, LATS2, ESR2, TRNT1, PLXDC1, CEACAM5, PRSS23, DUSP10, PDZD8, DNAL1, TLK1, PELI1, ERP29, FLJ22662, CPA3, MBL2 (upregulated) and FLJ36644, ZNF280B, CLASP2, TDG, IQCK, MPHOSPH9, NLGN1, NRIP1, FLJ10769, MLLT4 (downregulated) [177].

In normal breast, GR activity is necessary for milk production—Including ultrastructural changes in secretory cells, expression of milk proteins and inhibition of gland involution [178].

GRβ, the LBD-free form of GR, deserves special attention. It needs to be addressed that most studies treat GR as whole, disregarding the difference between receptors and thus disallowing conclusions on the exact action and relations of the isoforms.

GRβ was shown to stimulate the proliferation of LNCaP-ARA70β prostate cancer cells and to have a cross-talk relation with AR. It can be inhibited by mifepristone (as can be GRα) (2), in turn, ARA70β, an AR coactivator, has been found in MCF-7 breast cancer and PC3 prostate cancer lines and was shown to intensively stimulate the expression of NR3C1, which leads to preferential overexpression of GRβ [179]. GRβ acts as a dominant-negative inhibitor of GRα, causing glucocorticoid resistance (the other cause of that being the GRα gene loss or GRα phosphorylation) [170]. GRβ overexpression leads to less COX-2 suppression, excess cytokine (e.g., TNFα and IL-1) production and resulting enforcement of GRβ expression by NF-kβ pathway. GRβ potentiates insulin-dependent cell proliferation by suppressing PTEN and phosphorylating Akt1 in mice. The receptor expression can be induced by insulin or bombesin. In turn, GRα is repressed by Akt1, which phosphorylates its S134 residue [170] GRβ is preferentially expressed with AR in breast tumors and was revealed to be present in 92.1% of specimens, typically in triple-negative tumors. It has been shown that its presence is correlated with less vimentin expression in breast tumors in general and in non-triple-negative breast cancers and with lesser proliferation in breast cancers in general [175].

GR expression is positively correlated with ERα expression [180]. In infiltrative lobular carcinomas, these receptors are interdependent; GR suppresses growth, while ER promotes it. Estrogen stimulation leads to GR downregulation [170,180]. EREs often overlap with GREs and both hormones can affect the binding site of the other with various effects and the respective receptor remodels chromatin in specific sites, allowing the other to bind with them—Their combined presence (sometimes in a GR/ER complex) leads to increased interaction with AP-1, GATA and FOXO sites and to a more benign phenotype—Induction of VDR (cellular differentiation), KDM4B and others (chromatin remodelers), IGFBP4, CCDC88C (negative regulators of Wnt), repression of EMT genes (SNAI2, SOX2, EGR3, KLF9, TRERF1, SUV39H2, ARHGEF26, RHOU, RHOBTB1, ARHGAP36, TBC1D8, RET, DOCK4, CXCL12, LAMA3) [181]. The fact that GR and PR are sensitive to each other’s ligands and that GR and PR have about a half of their target genes shared between them is known; progesterone and glucocorticoids can bind to either GR or PR. For example, both induce upregulation of p21CIP1/WAF1 and phospho-p42/p44 (MAPK) protein in ABC28 cells, thereby reducing proliferation. The influence of GR on CTC15 cell morphology (grouping to form islets, cobblestone-like morphology) can be potentiated in presence of PR, whilst the effect is non-significant with PR alone or smaller with GR only. In T47-D cells both PR and GR are known to inhibit proliferation while activated. Activation of an unspecified variety of GR causes transition to S phase and DNA synthesis in CTC15 breast cancer cells [182]. Cross-talk between PR and GR causes growth inhibition and focal adhesion of breast cancer cells [180]. A study has shown no correlation of PR and GR expression in breast tumors [181].

Approximately 62% of primary invasive breast cancers express GRα [183]. GR mutates readily in many TNBCs [176]. Sporadic breast cancers exhibit BRCA1 downregulation correlated with tumor grade, rate of tumor progression, and risk of metastasis. It was shown that hydrocortisone leads to BRCA1 downregulation in normal breast cells. Unactivated GR binds to a RIBS element, co-acting with such transcription factors as GABPβ and Fra-2, thus indirectly upregulating transcription of the BRCA1 gene. GR and GABPβ interact by respectively the DBD and hinge region and N-terminal—central region. Mifepristone also exhibits similar influence on unactivated GR [184]. GR-positivity in ER+ breast cancers contributes to better outcome, and in ER- (triple negative) is related to shorter relapse-free survival [176].

In general, GR level in breast tumors decreases with progression. Worse long-term survival and more recurrence is expected in ER-negative tumors with greater expression of GR [176,183], however, in breast cancer overall GR expression is a sign of better outcome. GR expression was negatively correlated with tumor size, grade, pleomorphism, mitoses, lobular histological type, Ki67, CD71, AGTR1, p53, HER2 expression and HER+/ER+, basal P53 altered and basal P53 normal and triple negative phenotypes. It correlated positively with NPI good prognostic outcome, ER, PR, FOXA1, GATA3, BEX1 and luminal N and B subtypes [183]. Most of the correlations apply only for breast tumors overall and were not proven in ER+ subgroup. In ER- and TN tumors expressing GR, breast cancer specific survival was reduced, but was not an independent predictor of survival in Cox regression model; in other groups there was no association [183]. Another study, to the contrary, reported increased relapse-free survival for ER+ tumors expressing GR, regardless of the presence of PR [181].

GR target molecules induce F-actin and paxillin (focal adhesion proteins) expression and cell spreading in ABC28 cells and were evaluated to suppress their growth, on pair with aldosterone. Dexamethasone and aldosterone stopped the transition to S phase in mitotic ABC28 cells [182].

Glucocorticoids are antiproliferative towards breast cancer cells and reduce the inflammatory response to the tumor (according to several studies, breast tumor cells overexpress COX-2 which in turn causes prostaglandin production and other pro-proliferative changes—That were proven for immune cell tumors), therefore are used in treatment of primary breast cancer. In comparison, GR activation blocks apoptosis in normal breast epithelium by upregulating MKP-1. It was discovered that with tumor progression GR is translocated to the cytoplasm and its amount in the nucleus is significantly lower than in normal breast cells, surprisingly, this correlates positively with lower level of COX-2 (normally, GR represses COX-2). The exact effect of COX-2 on breast tumorigenesis is unclear, some propose COX-2 elevation as an event in the beginning of breast tumorigenesis, as their studies show its correlation with angiogenesis, lymphoinvasion and cancer progression, but other studies deny that. Cytoplasmic GR expression correlates with lower patient age of onset and with less disease-free time [180]. GRα hinders mitosis by upregulating p27 and p21. MCF-7 breast cancer cells present constant upregulation of GRβ and downregulation of GRα. 5-aza-2′-deoxycytidine (5-dAzaC), sodium butyrate (NaBu), and trichostatin A (TSA) introduction resulted in reversal of this ratio in several cell lines, including MCF-7 [170].

GRα expression seems to increase chemotherapy resistance and hinder apoptosis in TNBC and normal breast epithelium (via SGK1, MKP1/DUSP1, Forkhead factors and, presumably, reduced cleavage of caspase-3 and PARP); in case of tumors with high expression, this may happen even under physiological levels of cortisol. Introduction of mifepristone, a GR antagonist, with paclitaxel reverses resistance in MDA-MB-231, BT-20 cell lines and in murine MDA-MB-231 xenograft model—GR inhibition alone grants no such effect. A comparable effect was shown in MYC–overexpressing ER-negative MCF10A cell line [176]. In ER+ breast cancers, a therapy with a GR agonist is suggested [181].

## 7. Mineralocorticoid Receptor

MR, along with GR, are an evolutionary result of a gene duplication of a primitive corticoid receptor—with MR retaining the ability to bind aldosterone, 11-deoxycorticosterone and cortisol and GR being selective for cortisol only. Both associate with GREs when activated [26].

MR possesses the longest NTD of all steroid receptors [15,185].

NTD of MR contains five SUMOylation sites (K89, K399, K428, K494 in NTD, K953 in LBD) [15]. MR is SUMOylated by PIAS1 (protein inhibitor of activated STAT (signal transducer and activator of transcription), also known as SUMO-1 E3 ligase), however, the research on its influence yields conflicting results; it imparts no recognisable influence on its activity according to [186] but other studies have shown that SUMOylation leads to decreased transcription of normal GRE-related genes, but not of hemi-sites, degenerated GRE or MMTV promoter sites and that this relation is linearly correlated with the number of SUMOylated sites [15] MR dimers and heterodimers are especially prone to SUMOylation or poly-SUMOylation; SUMOylation may impair dimerisation of MR. SUMOylation can cause receptor translocation to the nucleus, however, it has not been proven to occur for MR. The exact effects of MR phosphorylation in humans are unknown, but those inferred from animal models or other steroid receptors include modulation of transcriptional activity, interaction with other proteins, modulation of its turnover and subcellular trafficking. Phosphorylation of Y73 in NTD in rats leads to significantly higher affinity of MR towards aldosterone and progesterone, similar effect (action enhancement) was noted for NTD-related phosphorylation by PKA (it is unclear whether PKA phosphorylates MR or its coregulator). Rapid phosphorylation of undefined serines and threonines is caused partly by PKCα and is related to ion transport. K367 in NTD and K715 in hinge are potential ubiquitinylation sites and are purported to increase receptor turnover and decrease its transactivation. Although MR binds Uba3 and Ubc12, it is unknown if its neddylation occurs. K677 of NLS1 is a potential location of MR acetylation by p300 or p/CAF and this modification is supposed to influence homodimerisation—GR heterodimerisation ratio and nuclear transport of the receptor [15].

The NTD of MR possesses neither the binding sequences present in the AR (FxxLF and WxxLF) nor those typical for all steroid receptors (LxxLL) for binding cofactors, thus the N/C-interaction of MR and cofactors is largely AF-2—independent [185]. MR activation is known to be mediated by several coregulators, seldom studied in comparison with other steroid receptors, although the conformation change may occur without cofactors, simply by binding aldosterone (Table 5) [186].

The chaperone proteins of MR include heat shock protein 90 (hsp90), which interacts directly with the receptor and other, acting indirectly, namely heat shock protein 70 (hsp70), p23, p48, FKBP-59 immunophilins, CYP40 cyclophillin and other immunophilins [15,187]. The exact composition of the complexes varies between different tissue types. The absence of hsp90 leads to deformation of the receptor’s structure and inhibition of steroid binding in NRs (it affects predominantly GR and MR) and the fluctuation of its concentration may lead to different transcriptional activity [190]. When not bound by a steroid, the MR forms a hetero-oligomer with a wide range of proteins, especially in the cytoplasm [187].

Target genes of MR include α/β/γ ENaC subunits, α1/β1-basolateral Na+,K+-ATPase pump subunits, CHIF, K-ras2, SGK1, ELL, GILZ (blocks ERK signaling), Grem2, Usp2-45, KS-WNK1 (worth of note because of further PI3K stimulation), NDRG2 (related to N-myc), ET-1, PAI-1, osteopontin, ACE, MDM2, EGF-R (indirectly: fibronectin), collagen I, III, IV (via ERK1/2), tenascin-X (TNX), ADAMTS1, hyaluronic acid synthase-2, RGS2, adrenomedullin, orosomucoid [187] and CNKSR3 [192]. MR blocks apoptosis via its competition for ELL, FAF and FLASH and induces BMP-2-like osteoblastic differentiation and mineralization [187].

Aldosterone exerts some rapid effects on tissues via AT-1 receptor. Active MR can induce SRC, EGFR and MAPK/ERK pathways, in case of the two latter by binding GPER and subsequent activation of EGFR and MEK/ERK. GPER is known to stimulate such pathways as MAPK/ERK, EGFR, PI3K/AKT, in addition to calcium mobilization and cAMP synthesis—resulting in cancer proliferation. By cooperation with HIF-1 GPER promotes VEGF expression and tumor angiogenesis. Various actions mediated by the GPER were assumed to be caused by its function as an aldosterone receptor, recently they are seen rather as the effect of binding with MR. Both MR and GPER are necessary for activation and increased expression of Na+/H+ exchanger-1 (NHE-1), a membrane protein involved in proliferation, migration and metastasis of breast cancer that acts in response to hypoxic, acidic tumor microenvironment. Furthermore, aldosterone caused K-RAS upregulation and Raf and Akt activation in renal carcinoma cells and activated another membrane protein, Na+/HCO3-cotransporter (NBC) [193]. MR acts as an inflammation promoter by upregulating ICAM1, while GR downregulates it [26].

The MR possesses several isoforms: the ‘classic’ MR and MR+4 variant with four additional aminoacids between the zinc fingers (caused by a cryptic splice site at the exon 3/ intron C splice junction), the hMRα and hMRβ (difference based purely on respective untranslated exons (1α, 1β), which both yield the same final protein and the only distinction between them can be shown in the mRNA phase as the different stability/efficiency in translation), the Δ5 or the Δ5,6 hMR isoforms (skipping exon 5 or exons 5 and 6) with greatly altered activity (eg. constant activity despite lack of steroids nearby). Two variants called MRA and MRB are caused by different start (Kozak) sequences chosen by ribosomes and are less prone to transactivation than the normal MR (with MRA more potent than MRB) [191]. Several other forms are known in mammals but are not proven in humans. In case of MR and MR+4, the latter isoform was once thought to be limited in presence to human white blood cells, but now has been shown to appear in various tissues, albeit in comparably smaller amounts than the classic one. There is no difference between their transcriptional activity. Both variants of MR are present in human breast tissue (in ratio of approx. 85:15) [15,194]. Several MR mutations are known in cancers (≥5% of colorectal cancer samples, in cutaneous melanoma, uterine, bladder, and stomach cancers, renal papillary cell carcinoma, glioblastoma multiforme), although none were reported as important for breast cancer development [26].

MR binds mainly aldosterone, although it may bind other mineralocorticoids and even glucocorticoids, based on the presence of 11β-hydroxysteroid dehydrogenase 2 in target cells (reduces cortisol to cortisone, thus disallowing competition) [15,194]. MR can heterodimerize with other steroid receptors, especially AR and GR [187]. The exertion of PR-like effects by the MR is suspected [175]. MR was shown to be expressed in larger amounts in presence of PR [175]. However, according to some sources, dexamethasone and deoxycorticosterone are weak agonists of MR in the absence of aldosterone, in its presence de facto working as antagonists by suppressing its N/C-interaction and consequent conformation change. Progesterone is stated as either ‘weak partial agonist but predominant antagonist’, a neutral ligand with no influence on MR in absence of aldosterone or downright antagonist in its presence [185]. Dexamethasone and aldosterone stopped the transition to S phase in ABC28 mitotic cells by blocking DNA replication. The absence of PR in MDA-MB-231-C2 cells led to markedly increased expression of MR. MR activation leads to meagre cell spreading in CTC15 cells [182].

Aldosterone hinders transcription of G6PD, mitochondrial uncoupling proteins UCP1 and UCP3, UPAR and HAS2 [187]. MR suppresses pyruvate kinase via induction of miRNA-186-5p, miRNA-95, and miRNA-338-3p, the last in turn suppresses PKLR (miR-338-3p-PKLR axis), thereby reducing Wartburg effect by disabling glycolysis, inducing lactate pathway and blocking hepatocellular carcinoma (HCC) proliferation (stopping cell cycle at G1 phase, downregulation of PCNA, apoptosis induction by CASP9 upregulation). Other targets of miR-338-3p include RAB14, SMO, P-Rex2a and SSX2IP (non-small-cell lung carcinoma, hepatocellular carcinoma, neuroblastoma and gastric cancer). MR downregulation was shown in 81.1% of samples of HCC and correlated with poor prognosis. MR exerted its effect by translocating into the nucleus, spironolactone partially blocked that transition, facilitated colony formation and blocked apoptosis. Deletion of 4q31.1 fragment hosting MR gene seems a peculiar trait of HCC, with 60% of samples presenting some type of MR loss [192]. In normal condition, PKLR, among other kinases, reduces autophagosome formation downstream or independent of mTORC1 in MCF-7 breast cells and in MCF-10A breast epithelial cells. PKLR silencing led to increased apoptosis despite optimal growth conditions in both lines [195].

The mineralocorticoid receptor (MR) has been shown to be present in both normal and cancer breast cells, with about 79–82.2% [196] specimens of tumors exhibiting the presence of MR [196], mainly in the cytoplasm [180]. The function of MR in breast tissue has been studied relatively poorly. Aldosterone was shown to elicit some influence on mammary gland development and differentiation and to potentiate prolactin-mediated casein synthesis in the breast of pregnant rabbits [193]. MR level does not seem to fluctuate during breast cancer progression [180]. The presence of MR correlates positively with triple negative breast cancer type, its cytoplasmic presence with node-negativity, more differentiated tumor cells and lesser proliferation and is related to reduced expression of vimentin and increased expression of SIP1 in TNBCs. Its presence reduced the relapse-free survival in non-triple-negative breast cancers (to more extent than tumor size and number of affected nodes), however, it led to better overall outcomes in triple-negative tumors that overexpress it (breast cancer specific survival, overall survival and distant disease-free survival). MR expression may lead to radiotherapy resistance in HER- cancers and to absence of distant metastases, it may induce epithelial-mesenchymal transformation—As of today this phenomenon has not been elucidated. Aldosterone was shown to induce growth suppression in ABC28 cells [182].

Apart from the treatment of cardiovascular diseases, MR can be exploited in cancer therapy. Prolonged (≥10 years) use of ARB/ACEi (angiotensin receptor blockers/angiotensin-converting-enzyme inhibitors) in Western populations significantly reduces breast cancer risk, it is disputed to be caused by suppressing renin-angiotensin II pathway and causing consequent downregulation of TF, VEGF, NF-κB and CREB. The mentioned drugs block adhesion and invasion of cancer cells by reducing production of integrin α3 and β1 subtypes and blocking VEGF-A (the latter proven only in mice) [197]. MR inhibitor (e.g., eplerenone, canrenone, spironolactone) use can be supportive in breast cancer chemotherapy, as it was proven to reduce cardiotoxicity and cardiovascular mortality of patients treated with anthracyclines—By reducing fibrosis, extracellular matrix turnover and myocardial collagen content and leading to better left ventricle parameters, notably lesser decrease of LVEF (left ventricular ejection fraction). A similar effect was shown for ACEi and ARB, although is disputed due to different heart failure criteria and differences in study groups. Candesartan, perindopril and lisinopril have shown a cardioprotective activity in breast cancer patients receiving trastuzumab and anthracyclines [198]. It is possible to insert cell cycle suppressory proteins like p53 (as plasmid) into cancer cells via liposomes harboring spironolactone; a study has shown that such measure leads to apoptosis in MCF-7 and MDA-MB-231 breast cancer cell lines, without toxicity to normal cells. The apoptosis is mediated by upregulation of BAX, cytochrome C and downregulation of Bcl-2 proteins [199]. MR downregulation by histone acetylation or methylation can be reversed by trichostatin A, sodium butyrate and 5-aza-2’-deoxycytidine in HCC lines [192].

## 8. Vitamin D Receptor

The information on different isoforms of VDR is scarce. Ten proven isoforms and two putative are known. It is supposed that their expression is tissue-related and that they have different target gene profile, although it has not been elucidated properly as of now [200,201].

VDR is more stable upon binding an appropriate ligand [200]. VDR canonically homodimerizes with other VDR [202] or heterodimerizes with RXR when activated or attaches to a binding site on DNA with pre-bound RXR (the number of these sites increases in osteoblasts after introducing D3), other combinations are also known (VDR-THR, VDR-RAR). In the absence of ligands, VDR binds corepressors [203,204]. Regarding VDR-RXR heterodimers, the studied cofactors include CREB-binding protein/p300, steroid receptor coactivator family and SWI/SNF complexes. The RUNX2 and C/EBPβ also seem to have their actions correlated with VDR [205]. PPARG, BCL6, ESR1, RUNX1, STAT1, MYC and CYP24 seem to be activated by bound VDR and EGR1, SP2, ZNF and STAT1 by free VDR [204]. VDR influences the expression of ITGβ3, SLC1A1, KDR, BIRC3 and GLUL, exerting an antiproliferative phenotype, promotes CYP24A1, SERPINB1, EFTUD1, CLMN, KLK6—The three latter are related to better survival in breast cancer [200]. VDR phosphorylation of S208 by casein kinase II seems to lead to increased transcriptional activity, its activity may be decreased by phosphorylation of dimeric partners (RXRα, S260 by Ras/ERK). Other coregulators of VDR include Smad3, TFIIB and c-Jun [202]. VDR expression can be decreased by Ras/ERK pathway (though MEK1, a factor stimulating this pathway, exerted no effect on VDR transcription), either by direct transcription repression, VDR-RXR dimer disruption or p38/MAPK inhibition. p38 and JNK, activated by MKK6 or MEKK1, mediate VDR gene transactivation via c-Jun/AP-1. AP-1, in turn, comprises homodimers of Jun (c-Jun, JunD, JunB) or heterodimers of Jun with Fos (c-Fos, FosB, Fra-1, Fra-2) or others like ATF2, CREB and NFAT. This upregulation leads to reduced growth of MCF-7 cells. MKK6 activity can be substituted with arsenite [202]. Additionally, vitamin D receptor can be corepressed by SIN3/NurD/CoREST, PRC2, TFTC, and SWI/SNF and coactivated by complexes such as INO80, CBP, and SRC3/ncoa3 and WTAP-SFRS [204]. Actually, calcitriol itself can increase the expression of VDR [200,201].

VDR displays some peculiar differences caused by polymorphism of its gene—With the most studied mutations known as Cdx2, Fok1, Bsm1, Apa1, Bgl1, Taq1, and Poly (A). According to [206], codominant models such as Bsm1 bb, Apa1 aa, Poly (A) LL, recessive contrast models of Fok1, Apa1, and Poly (A) and allelic contrast model Poly(A) L are correlated positively with occurrence of breast cancer. There is no proven relation between Cdx2, Bgl1, Taq1 and breast cancer (however, some studies have proven a putative connection with Cdx2) [206].

Vitamin D receptor is expressed by normal lobule, ductal epithelial cells [5], stromal and immune cells [203], adipose cells [200] of human breast and in a higher rate in breast cancer lesions (though there exists some data on the contrary) and in invasive cancers, as compared to in situ cancers or normal tissue [5]. VDR is responsible for breast cell differentiation (ductal differentiation and branching) [201], pubertal development, late pregnancy changes and lactation, calcium transport, casein expression [200] and controlled the menstrual cycle-dependent morphological changes of normal breast tissue in mice [204]. VDR paracrine action between adipose and epithelial breast cells might be important in its normal development and oncogenesis. VDR expression in breast is promoted by lactogens and can be influenced by miR-125b [200]. It may be present intranuclearily, intracytoplasmally and on either cell or nuclear membrane. Cytoplasmal, unactivated VDR promotes proliferation and nuclear, bound form hinders it [5]. Breast tumors in mice are known to accumulate 25-OH-D (incorporating bound DBP by megalin-cubilin complex), however, its significance and presence of this phenomenon is unknown in humans. Nevertheless, breast tissue in humans can synthesize D3 from 25-OH-D by CYP27B1 present therein or acquire it from breast adipose cells, harboring CYP27B1. CYP27B1 expression was reduced in immortalized human breast epithelial cells, inhibiting the actions of 1,25-OH-D and similar effect (upregulated cell proliferation, angiogenesis, cell cycle progression, survival markers in tumors, elevated oncogenic pathways like AKT, NF-κB and STAT3) is known for murine PyMT-MMTV model. Presence of such effect in native human breast tumors is unclear. CYP24A1, which reduces 1,25-OH-D to 25-OH-D, was shown to be present in 50% of breast cancer samples, whereas its expression is low in normal breast tissue (excluding luminal epithelial progenitors). The elevation of CYP24A1 is highly heterogenous and might either have no effects (as a cell possesses high levels of VDR, e.g., MCF-10A, MCF-7, cultivated normal epithelial lines) or promote growth and tumorigenesis (MB-MDA-231). In general, the response of cancer cell lines to 1,25-OH-D is varied and dependent on their specific genome alterations, not on the VDR itself [200].

Although it was shown that breast cancer cells have elevated expression of VDR, they produce exotic receptor variants (V3, V1d″) and less full-length variants (V1, V2, V1d, V1d′); truncated, downstream aberrant variants (yet unnamed) contribute to much of the VDR-related mRNA production. The V1 and V2 suppression may be caused by promoter hypermethylation [200,201].

VDR is rarely mutated in cancers, albeit can be disabled by aberrant histone deacetylation and methylation of its gene. VDR promoter region (−760–−480), 1a and 1d exons of VDR contain several parts (including three CpG islands) prone to methylation and overlapping with SP1, AP-2, NF-κB, and transcription start sites. In spite of the fact that methylation occurs in any normal breast samples, breast cancer cell lines (HS578T, 21PT, MCF7, T47D) and breast cancer samples exhibited elevated VDR promoter region methylation and significant hypermethylation of CpGs (40–65% dinucleotides), as compared to immortalized normal breast line (HBL100) or normal breast samples. In fact, normal breast samples employ meagre levels of methylation (av. 15%) as compared to breast cancer samples (av. 65%). Demethylation of these sites by 5′deoxy-azacytidine led to decreased cell viability in response to calcitriol in all breast cell lines and to increased expression of VDR, β-actin, p21, C/EBP, CYP27B1 and CYP24A1 [201]. CYP27B1 silencing had similar pro-proliferative effect as VDR silencing as is known to upregulate AKT, NF-κB and STAT3 pathways, associated with progression [200].

The relation of ER and VDR transcription is unclear: some studies show their coexpression in breast cancer samples and note their related stimulation effects [207], however, other studies either discovered that VDR expression induction does not cause parallel increase of ER [202]. VDR coexpression with AR and ER in cancer-surrounding breast tissue contributed to more favorable outcomes [5]. The research concerning the relation of VDR and ERβ is, as of now, scarce. A notable apoptotic, pro-differentiative and antimitotic cross-talk between AR and VDR can be utilized in therapy [134]. Knockout of VDR in mice led to enhanced breast gland growth response to estrogen and progesterone in vivo.

VDR was expressed in considerable amounts in 82.9–91.9% [5,203] of invasive breast cancers. Breast tumor tissues stained for VDR ‘almost exclusively’ as compared to their surroundings; invasive tumors showing more intense expression of VDR and more nuclear-centred, in situ tumors with cytoplasmic staining [5]. Vitamin D is an antiproliferative agent in breast cancer [203]. The absence of VDR correlated positively with greater tumor size, high Nottingham grade, absence of ER or PR, high expression of Ki67 [5] and lymph node involvement [203]. VDR loss dominates in luminal B-like and triple-negative tumors (with percentage of respectively 25.6% and 78.4% of tumors). HER2 negativity also occurs concurrently with low levels of cytoplasmic VDR [203]. Higher VDR levels correlate with higher ER levels in cells [207], with a more benign phenotype and longer relapse-free survival [181]. The presence of VDR precludes less occurrences of mastectomy and lower risk in breast cancer related death in affected patients in general (regarding molecular subtypes of breast cancer, a correlation of less mortality has been proven for luminal B-like tumors and is putative for luminal A-like tumors, while non-significant in triple-negative or basal cancers) [5]. Vitamin D has been found to induce autophagy in luminal-like breast cancer cells (MCF-7, MDA-MB-453, ZR-75-1) and normal stromal breast cells, mainly by upregulation of MAP1LC31B, Beclin1 and a set of other proteins related, however, the same effect appeared after VDR knockout [204]. The progression-free survival length is also related to the expression of VDR (with a difference of about 6 years between the groups of patients with low and high expression) [203]. However, studies show conflicting data on the influence of the exact level of the receptor’s expression on mentioned parameters [5,203], some even stating no significant influence of VDR expression on the outcome [207]. VDR expression correlates positively with positive outcomes in luminal A breast cancer, especially in cases treated with tamoxifen—probably due to higher ER levels that correlate with more benefit form tamoxifen use [207]. VDR expression halts in highly aggressive tumors [200]. Concluding, VDR expression is related to longer progression-free survival, and overall survival in univariate analyses [5].

The risk of death is equally elevated in patients with not only reduced, but also elevated level of seral vitamin D [5]. The debated influence of D3 on breast cancer proposes some answers to breast cancer statistics: as both VDR and CYP27B1 upregulate during lactation [200], it may be the factor contributing to positive influence of breastfeeding on breast cancer incidence [208]. Similarly, lower levels of D3 after menopause [209], in obese patients [210] and in patients of African or Asian descent living in moderate climate [211] may all result in higher incidence of breast cancer [212].

Vitamin D induced apoptosis via expression of autophagy marker LC3 (LC3A/B—despite its suppression by inactive VDR), Beclin1, via calcium/calmodulin-dependent protein kinase kinase 2 (CAMKK2) and subsequent AMPK activation [204]. Its apoptotic expression profile appears mostly in normal breast cells and reverses with cancer progression and invasion. For example, in MCF-7 cell line, EGFR, ITPR1, MAP1LC3B, SQSTM1, SAR1A, PRKAR1A and SESN2 were downregulated by vitamin D and PTK6, CLN3, CXCR4, PRKCD, RAB1A, CAPN1, CDKN1B, GNAI3, DRAM1, INSR, ARNT, ZFYVE16, RAB11A—upregulated, while invasive ductal and lobular breast carcinoma cells reacted otherwise. The downregulation of CALCOCO2, KRT13, SAR1A, RAB11A and MAP1LC3B correlated with poorer survival. The same seems to happen to the normal stromal cells, with VDR-related upregulation of HSP90AB1, PRKAR1A, MAPK9, SQSTM1, HIF1A, SAR1A, TGM2, ITPR1 and EGFR, downregulation of DRAM1, CXCR4, ULK2, NRG3, WIPI1, TNFSF10, KLHL24, PI3KC3, ESR1, ATG12, PRKCD, GABARAPL2, CALCOCO2, CAPN1, BNIP3L, RHEB, PTEN, CLN3, PRKAA1, ZFYVE16 (the stroma of invasive cancer repeats the pattern of opposite effect, seen in carcinoma cells) [204]. In MCF-7 the most upregulated genes by calcitriol were respectively CYP24A1, SHE, CRYBB3, TRPV6, ROPN1B, IGHE, ZFP92, RNF222, PRKG2, SH3TC1, IQUB, GOLGA2P2Y, SBK2, NPPC, SLC37A2, SEMA6D, TMPRSS2, MERTK, KLK6, LOC389602 and SNAI2 and the most important target functions are stated as ‘pathways involved in muscle cell migration, protein citrullination, negative regulation of cellular processes, negative regulation of locomotion, neuron development and citrulline biosynthetic processes (...) bone morphogenesis, ossification and eyelid development’ [207].

BT-20, MDA-MB-468, SUM-159PT, MFM-223, CAL-148, ZR-75-B, and BT-474 cell lines are all susceptible to VDR-induced apoptosis. BT-549, a notable exception, possesses no VDR [134].

Inecalcitol, a vitamin D analog with less calcaemic potency, was shown to be eighteen times more effective as vitamin D in apoptosis induction in breast cancer cells, prompting growth halt even in VDR-deficient breast cancer cell lines resistant to vitamin D. VDR activation promotes apoptosis more easily in ER+ lines. This effect is independent of the cells’ AR or HER2 status. Both compounds reduced cell migration (in MCF-7, BT20, Hs578t(i8), in MDA MB 453—only inecalcitol). Apart from calcitriol-related genes, inecalcitol stimulated CYTH4, LOC553137, MYBPH, GPR78, FREM1, B4GALNT2 and showed preferential upregulation of CLMN, CYP24A1, TMRPSS2, ARHGEF6, FOS, IGFBP3, PISD, G6PD, ITPR1, KLK6, SHE, ACOX3, FSTL4, MERTK and PADI3 [207]. Forskolin and phorbol 12-myristate 13-acetate are also known to unspecifically increase VDR level in cells [202].

As TNBCs do express VDR or AR (about 2/3 of tumors) and some ER+ tumors gain resistance to targeted therapy, they can be treated with VDR agonists (e.g., seocalcitol), especially coupled with AR agonists and conventional chemotherapy. This type of treatment leads to G1 phase arrest, increase of apoptosis, reduction of tumorsphere formation efficiency and cancer stem cell features (by deactivation of CD49f, SOX2, and Notch pathways), with epithelial transformation (increase of claudin-4, cytokeratin 18, down-regulation of cytokeratin 5 and vimentin). In this case, vitamin D analogues are preferred to avoid side effects of normal vitamin D in high concentrations [134].

## 9. Conclusions

Most patients in the advanced stage of the neoplastic disease spread and consequently develop numerous symptoms, which is the basis for considering systemic treatment (chemotherapy, hormone therapy, immunotherapy, targeted therapy). The most common indications for hormone therapy include cancers whose growth is dependent on estrogens (breast cancer) and progestins (endometrial cancer) in women and testosterone in men (prostate cancer). Reduction of the effect of hormonal factors on tumor cells can be achieved by inhibiting the synthesis of sex hormones, administering analogues of the luteinizing hormone releasing factor in the pituitary gland (goserelin, leuprorelin), blocking peripheral synthesis (aromatase enzyme inhibitors) or by limiting the direct peripheral influence of sex hormones on receptors located on tumor cells (antiestrogens, antiandrogens). Contrary to chemotherapy, which causes numerous side effects, hormone treatment is much better tolerated and is often continued for the rest of the patients’ lives. However, it is important to be aware of the limitations of hormone therapy, which include the subsequent therapeutic response, side effects and drug interactions [213].

The huge therapeutic potential of the SRs together with possible risks associated with endocrine therapies yield a need for constant improvement. Areas to explore include e.g., targeting PR depending on the ratio of isoforms, application of AR antagonists in AR+ TNBC, use of MR antagonists to prevent cardiotoxicity associated with chemotherapy or targeting VDR with vitamin D analogues such as inecalcitol. Given the immense complexity of SRs function in breast cancer, which provides multiple potential therapy targets, it is easy to expect that in the near future many more novel concepts on therapeutic applications of steroid receptors will arise.

## Figures and Tables

**Table 1:** Coregulators of PR transcriptional action [30,84]. Molecular function of the proteins as stated in the UniProt online database https://www.uniprot.org/ [91]. Depending on the molecular context the BRG1 protein can act as a coactivator (for example serving as a component of the SWI/SNF complex) or as a corepressor [84,91].

Coregulator Type	Gene (Protein) Name	Molecular Function
Coactivator	NCOA1 (Nuclear Receptor Coactivator 1) also known as SRC1	Acyltransferase, activator
	NCOA3 (Nuclear Receptor Coactivator 3) also known as SRC3	
	EP300 (Histone acetyltransferase p300)	
	CREBBP (CREB-binding protein)	
	NCOA2 (Nuclear Receptor Coactivator 2) also known as SRC2, TIF2 or GRIP1	Activator
	Components of the SWI/SNF (Switch/Sucrose Non-Fermentable) complex	Helicase, DNA-binding, hydrolase, chromatin regulator
	UBE3A (Ubiquitin-protein ligase E3A) also known as E6AP	Transferase
	CARM1 (Histone-arginine methyltransferase CARM1)	Methyltransferase, chromatin regulator
	SRA1 (Steroid receptor RNA activator 1)	Receptor, activator
Corepressor	NCOR1 (Nuclear receptor corepressor 1)	DNA-binding, chromatin regulator, repressor
	NCOR2 (Nuclear receptor corepressor 1) also known as SMRT	DNA-binding, repressor
	HDAC1 (Histone deacetylase 1)	Hydrolase, chromatin regulator, repressor
	HDAC2 (Histone deacetylase 2)	
	RCOR1 (REST corepressor 1)	Chromatin regulator, repressor
	CBX3 (Chromobox protein homolog 3) also known as HP1γ	
	KDM1A (Lysine-specific histone demethylase 1A) also known as LSD1	Oxidoreductase, chromatin regulator, repressor
Coactivator/corepressor	SMARCA4 (Transcription activator BRG1)	Helicase, hydrolase, chromatin regulator, activator, repressor, RNA-binding

**Table 2:** Phosphorylated residues of the AR and their functional roles. Reprinted from the paper “Posttranslational Modification of the Androgen Receptor in Prostate Cancer” by Van der Steen, Tindall and Huang; published in International Journal of Molecular Sciences (publisher: MDPI), 2013 [128].

Residue	Kinase/Phosphatase	Function	References
S81	CDK1, CDK5, CDK9	Localization, protein stability	[144]
	PP2	Cell growth, transcription	[145]
S94	PP2	Transcription	[145]
S213	PI3K/AKT1	Localization	[146,147,148]
	PIM-1	Stability	[144,149]
Y267	Ack	Cell growth, transcription	[150,151,152,153]
	SRC		
T280/S291	AurA	Cell growth, transcription	[154]
S308	PP2	Transcription	[145]
Y363	Ack	Cell growth, transcription	[151]
S424	PP2	Transcription stability	[145]
	PP1		
S515	MAPK	Transcription, degradation	[155,156]
	CDK7		
Y534	SRC	Localization, cell cycle, transcription	[153,157]
S578		Localization, transcription	[156]
S650	ERK1/JNK1/p38-alpha	Localization	[158]
		Transcription	[159]
	PP1	Localization	[160]
S791	PI3K/AKT1	Transcription, apoptosis, localization	[146,147,148]
T850	PIM-1L	Stability	[161]

**Table 3:** The interactome of AR (citation: if not marked—[162]).

Protein Type	Activation-Associated	Suppression-Associated
Components of the chromatin remodeling complex	ARIP, BRG, hBRM, BAF57, SRG3/BAF155, SRCAP, hOsa1/BAF250, hOsa2	
Chromatin structure	HMG-1, HMG-2	
Acetyltransferases and deacetylases	NCOA1 (SRC1) [163], NCOA2 (SRC2), NCOA3 (SRC3) (Rac3, ACTR, AIB1, p/CIP, TRAM1) [163], p300, CBP, P/CAF, Tip60 [162,163]	HBO1, SIRT1, HDAC7, other HDAC
Methyltransferases and demethylases	CARM1/PRMT5, PRMT1, G9a, NSD1/ARA267α, LSD1, JHDM2A, JMJD2C [162], KDM4A, KDM4D, KDM4C, KDM4B [128]	
Ubiquitination/proteasome pathway	E6-AP, PIRH2, SNURF/RNF4, ARA54, USP10, UBCH7 [162], ZIPK [128]	Mdm2, Chip, MKRN1
	TSG101 (both groups), ARNIP (no data)	
SUMOylation pathway	SUMO-2, SUMO-3, Ubc9, Zimp7, Zimp10, SENP1	SUMO-1, PIASy, Uba3
	PIAS1, PIAS3, PIASxα/ARIP3, PIASxβ	
Splicing and RNA metabolism	p54nrb, p102 U5snRNP/ANT-1, p44/MEP50	hnRNPA1
	PSF, PSP1, PSP2 (no data)	
DNA repair	Ku70, Ku80, DNA-PKc, BRCA1, BRCA2	Rad9
Chaperones and cochaperones	Hsp40 (DnaJ, Ydj1p), Hsp90, Hsp70, Cdc37, FKBP52, FKBP51, Bag-1L	DjA1
Cytoskeleton	actin, supervillin, gelsolin, filamin, α-actinin-2	filamin-A, transgelin, ARA67/PAT1/APPBP
	α-actinin-4	
Endocytosis	HIP1, GAK/auxillin2, caveolin-1	APPL
Signal integrators and transducers, scaffolds and adaptors	ARA55 (Hic5) [163], paxillin, FHL2 (DRAL) [163], PELP1/MNAR, vinexin-α, Vav3, Rho GDI, Ack1, PRK1, RanBPM, ARA24/Ran, STAT3, β-catenin [162], calreticulin [163]	PAK6, RACK1, Ebp1, Hey1, Hey2, RNase L, TCF4
	Smad3, GSK-3β	
Cell cycle regulators	cyclin E, cdc25B, CDK6, Rb, pp32, RbaK, AATF/Che-1 [162], RAF (IDE) [163]	cyclin D1
Regulators of apoptosis	Par-4	caspase 8
Viral oncoproteins	E2, Hbx	
	E6, E7	
Nuclear receptor coregulators	Asc-1, Asc-2, Trap/Mediator complex proteins, CoCoA, NRIP, PNRC, TIF1-α, MRF1, PDIP1, Zac1, GT198, ARA70 (RFG, ELE1) [163], ART-27, ARA160 (TMF) [163], PGC-1 (LEM6), NCOA2 [163]	Alien, AES, SMRT, NCOR, PATZ, TGIF, TIP110, TZF, ARR19
	RIP140 (depending on receptor-coregulator ratio—corepressor in high, coactivator in low) [163]	
Kinases and phosphatases	MAK, ANPK, Dyrk1A, RSK	ERK8, SCP2, PP2A
Transcription factors	AML3/CBFα1, EGR1, FOXA1, GATA-2, GATA-3, NF1, PDEF, Sp1, SF1, USF2, SRF [164], FOXO4	AP-1, ATF2, c-rel, c/EBPα, Dax1, ERα, FKHR, FoxH1, GR, HoxB13, Pod-1, p53, RelA, SRY, SHP, TR2, TR4
	Brn-1, c-Jun, Foxa2, Oct-1, RXR, Sox9, Oct-2 (no data)	
Other	DJ-1/PARK7, L-dopa-decarboxylase, MAGEA11, SRA	LATS2/KPM, PTEN, Tob1, Tob2, DJBP

**Table 4:** Sample elements of GR interactome and the modifications of the GR [171,172,173].

Protein Feature		Protein Name
GR interactome	Activation-associated	HSP90, p23, FKBP51, FKBP52, Cyp44 [171], PP5 [171], HDAC6, REV-ERBα—protection, activation, translocation
		MAPKs, CDK, GSK3 (by phosphorylation)
		CBP, P300, PCAF, p160 family: NCOA1, NCOA2, NCOA3 [171,173]—histone acetyl transferases
		Mediator complex—MED1 and MED14
		C/EBPα, C/EBPβ, COUP-TFII, CREB1, E47, FOXA1, FOXA2, FOXO1, LXRβ, HNF6, PPARα, BMAL1, CLOCK, CRY1/CRY2, PER1/2, REV-ERBα/β, RORα/γ, HNF1α, HNF4α, STAT5, FXR—transcription factors
		AP-1, AP-2, NFB, NF‐κB, ER, CREBP, NF1/CTF1, Yin Yang 1, Sp1, IRF1/2, cMyb, PU.1, EGR1/NGF1-A [171], 14–3–3ζ, 14–3–3η
		CRTC2, SIRT1, PGC-1α, ASCOM complex, SETDB2
	Suppression-associated	SMRT, HDAC1, CtBP, SMAD6-HDAC3, CRY1, TAZ, NCOR [173]
		LXRα—transcription factor
		14–3–3σ, FLASH, G protein β [171]
		GC responsive factor-1, c-Ets-1/2 [171]
	Deactivated by GR	MAPK, PI3K, TCR complex [171]
GR modifiers [171]	Phosphorylation	CDKs (A-CDK2, A-CDC2, B-CDK2, B-CDC2, E-CDK2, CDK5), p38 MAPKs, AKT, JNKs, GSK-3β, ERK, casein kinase II
	Dephosphorylation	PP1, PP2a, PP5
	Ubiquitination	E-1, E-2 (UbcH7), E-3 (Hsp70-interacting protein, ET-AP, human homolog of mdm2 (hmdm2) + p53)
	SUMOylation	Ubc9, RSUME
	Acetylation	CLOCK, BMAL1
	Deacetylation	HDAC2
	Nitrosylation	neuronal NO synthase, NO donors
	Oxidation	H_2_O_2_
	Reduction	dithiothreitol, N-acetyl-L-cysteine, thioredoxin reductase

**Table 5:** Interactome of MR (citation: if not marked—[186]).

Protein Feature	Protein Name	Interaction Site
Activation-associated	ELL (elongation factor)	NTD [185], AF-1b [186,187]
	SUMO-1-conjugation enzyme (Ubc9)	NTD
	NCOA1 (SRC-1)	AF-2, NTD [187]
	SRC-1e isoform	AF-1, NTD [187]
	NCOA2	AF-2 [187]
	p300/CBP	AF-1 [186], AF-2 [186,187], NLS1/hinge? [15]
	PGC-1α	AF-2 [186,187,188,189,190], LBD [188]
	PGC-1β	LBD [188]
	ASC2 (NCOA6, RAP250, AIB3, PRIP, TRBP, NRC), ASC2-1	LBD [188]
	CREBP-BP/RNA helicase complex	NTD
	FLASH	AF-1, NTD [187]
	CIA	LBD [188]
	ARA70-1, ARA70-2	LBD [188]
	FAF-1	AF-1, NTD [187]
	TIF1 [187], TIF1α	NTD
	NSD1	LBD [188]
	RIP140	NTD
	Tesmin	LBD
	RHA (RNA helicase A) [185]	AF-1a [187], NTD [191]
	GAL4 response element [185]	DBD [186]
	PKA [15]	NTD
	p/CAF [15]	NLS1/hinge (probably p300-related)
	EEF1A1, XRCC6, other MR dimers [185], EIF5B [189], AGAP002076-PA—similar [189], PKCα [15], Uba3, Ubc12, NEDD [15], HDAC [187]?	?
Suppression-associated	PIAS1	NTD/LBD [187]
	SMRT	LBD
	NCOR	LBD
	DAXX	NTD
	NF-YC	AF-1
	Gemin 4	LBD?
	PIASxβ [187]	NTD?
	SSRP1 [186,189], ATRX [189], SAFB [189], AHNAK [189], BUB3 [189], NPIPL3 [189], CCDC55 [189], RPL4 [189], XRCC6 [189], RRBP1 [189], RPL23A [189], SERF2 [189], EEF1A1 [189], ENSA [189], MUC1 [189], Chloride intracellular channel 1 variant [189], GPX3 [189], RRBP1 [189], PCBP2 [189]	?
	MYL2 [189], FRMD4B [189]	?
Chaperones	hsp90	LBD
	hsp70, p23, p48, FKBP-59, CYP40, other immunophilins	indirectly, via Hsp90
	actin [187]	LBD
